# Thermosensory TRPV Heterotetramers Drive Seasonal Polyphenism: Molecular Basis of CcIav/CcNan‐PKCα‐AKH/AKHR Signaling in Pear Psyllid Morph Transition

**DOI:** 10.1002/advs.202510102

**Published:** 2025-09-15

**Authors:** Jianying Li, Zhixian Zhang, Yilin Wang, Yue Yang, Jiarui Wang, Xueyi Ruan, Songdou Zhang

**Affiliations:** ^1^ Plant Protection Research Institute Guangdong Academy of Agricultural Sciences Key Laboratory of Green Prevention and Control on Fruits and Vegetables in South China Ministry of Agriculture and Rural Affairs, Guangdong Provincial Key Laboratory of High Technology for Plant Protection Guangzhou 510640 P. R. China; ^2^ Department of Entomology and MOA Key Lab of Pest Monitoring and Green Management College of Plant Protection China Agricultural University Beijing 100193 China

**Keywords:** adipokinetic hormone, cacopsylla chinensis, protein kinase CcPKCα, seasonal polyphenism, TRPV channels

## Abstract

Temperature critically governs seasonal polyphenism, shaping species distribution and invasion dynamics. Although vanilloid‐type transient receptor potential (TRPV) channels are conserved thermal sensors, their mechanistic role in orchestrating seasonal polyphenism remains unresolved. The pear psyllid *Cacopsylla chinensis* exhibits striking temperature‐dependent transitions: warm spring temperature triggers winter‐form adult females to oviposit and irreversibly transform into summer‐forms, providing a model to dissect this phenomenon. Here, how TRPV‐mediated thermal sensing regulates this process. It is demonstrated that exposure to 25 °C – a critical ecological threshold‐induces an orchestrated shift in energy metabolism, follicular epithelium remodeling, and ovarian maturation, culminating in phenotypic transformation. Central to this process is the assembly of functional heterotetrameric TRPV channels comprising interdependent subunits CcIav and CcNan, which directly perceive the 25 °C thermal signal. Mechanistically, CcIav/CcNan activation triggers a phosphorylation cascade via protein kinase CcPKCα, subsequently potentiating adipokinetic hormone signaling through CcAKHR. This axis synchronizes energy metabolism reprogramming with reproductive tissue reorganization to drive polyphenic switching. Significantly, the evolutionary conservation of this TRPV is validated heterotetramer‐dependent thermosensation mechanism across three phylogenetically diverse insect species. This work delineates a previously unrecognized CcIav/CcNan‐CcPKCα‐AKH/CcAKHR signaling module essential for insect seasonal adaptation and pioneers a receptor‐targeted strategy for disrupting psyllid population cycles in agroecosystems.

## Graphical abstract

At 2 5 °C, thermal activation initiates: i) CcIav/CcNan heterotetramer assembly → Ca^2^⁺ influx; ii) CcPKCα phosphorylation → AKH signaling potentiation; iii) CcAKH1 binding to membrane‐localized CcAKHR → energy mobilization (lipid catabolism/glycogenolysis) and vitellogenin transport via follicular patency; iv) Oocyte maturation → summer‐form nymph production and population establishment.

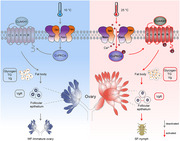

## 1 Introduction

Polyphenism – the capacity of a single genotype to produce discrete phenotypic outcomes in response to environmental signals – represents an evolutionary triumph that enables organisms to colonize fluctuating habitats.^[^ ^1^ ^]^ Notable examples of polyphenic adaptations span both invertebrate and vertebrate lineages, including: i) temperature‐driven seasonal polyphenism in insects such as the pear psyllid *Cacopsylla chinensis* ^[^ ^2^, ^3^ ^]^ and butterflies^[^ ^4^ ^]^; ii) caste differentiation in eusocial insects mediated by social stimuli^[^ ^5^ ^]^; iii) environment‐dependent sex determination systems in reptiles^[^ ^6^ ^]^ and teleost fish^[^ ^7^ ^]^; and iv) developmental plasticity in plants responding to abiotic stressors.^[^ ^8^ ^]^ *C. chinensis*, a vector of the devastating pear tree disease fire blight, showcases notable morphological disparities between its summer‐ and winter‐forms in response to seasonal changes.^[^ ^9^, ^10^ ^]^ This psyllid's cold‐adapted winter‐form enters reproductive diapause with thickened cuticles, while spring warming triggers ovarian activation and irreversible transformation into the summer‐form. Intriguingly, while the temperature receptor *CcTRPM*‐mediated cold‐sensing pathway driving summer‐to‐winter transition has been elucidated,^[^ ^2^ ^]^ the thermal sensors governing the reverse process remain enigmatic – a critical knowledge gap given the pest's expanding range under climate change.

The transient receptor potential (TRP) superfamily, encompassing diverse cation‐permeable channels, is phylogenetically classified into seven subfamilies: TRPC (canonical), TRPV (vanilloid), TRPM (melastatin), TRPP (polycystins), TRPML (mucolipins), TRPA (ankyrin), and TRPN (no mechanosensor potential C).^[^ ^11^, ^12^ ^]^ The TRPV subfamily, evolutionarily conserved thermosensors in metazoans, forms functional heterotetramers through combinatorial assembly of subunits like Inactive (Iav) and Nanchung (Nan).^[^ ^13^, ^14^, ^15^ ^]^ Unlike mammals, where TRPV1‐6 homomers dominate the thermosensation, insects rely exclusively on Iav/Nan heteromers – a dichotomy with profound mechanistic implications.^[^ ^16^, ^17^, ^18^ ^]^ Recent phylostratigraphic analysis reveals striking sequence divergence in insect TRPVs (<30% identity across taxa), suggesting lineage‐specific adaptations in thermal sensing.^[^ ^19^, ^20^ ^]^ Crucially, these heteromeric channels interface with downstream effectors: protein kinase C (PKC) phosphorylation gates TRPV activation,^[^ ^21^ ^]^ while adipokinetic hormone (AKH) signaling integrates metabolic and reproductive states.^[^ ^22^ ^]^ Yet how these components coalesce into a temperature‐responsive network remains unexplored.

Central to seasonal morph transition is the metabolic overhaul of diapause termination.^[^ ^23^ ^]^ The bioenergetic regulation underlying diapause termination‐ particularly the coordination of reproductive activation with development transition‐remains poorly understood. AKH, an insect‐specific neuropeptide secreted by the corpora cardiaca that governs energy homeostasis through receptor AKHR signaling. Beyond its canonical role in lipid mobilization,^[^ ^22^ ^]^ AKH signaling integrates diverse physiological programs including reproductive plasticity, metamorphic timing, immune priming, and stress resistance.^[^ ^24^, ^25^ ^]^ Striking functional conservation emerges across taxa: AKH induces vitellogenesis and oocyte maturation in *Locusta migratoria*,^[^ ^26^ ^]^ modulates vitellogenesis and nutrient storage in *Aedes aegypti*.^[^ ^27^, ^28^ ^]^ Pathogen manipulation of this system is exemplified by “*Candidatus* Liberibacter asiaticus”, which hijacks AKH‐mediated lipid droplet dynamics and triglyceride levels to enhance *Diaphorina citri* fecundity.^[^ ^29^ ^]^ Importantly, successful vitellogenesis requires vitellogenin (Vg) transcytosis across the follicular epithelium—a process facilitated by transient intercellular patency formation.^[^ ^30^, ^31^ ^]^ This dual demand – metabolic activation synchronized with ovarian restructuring – implies exquisite coordination between environmental sensors and endocrine effectors. We hypothesize that TRPV heterotetramers serve as thermal integrators linking temperature cues to AKH‐driven physiological switching.

Here, we dissect the 25 °C‐triggered signaling cascade driving *C. chinensis* winter‐summer transition. Through calcium imaging, co‐immunoprecipitation, immunofluorescence, qRT‐PCR, and RNAi, we establish CcIav/CcNan heterotetramers as bona fide warmth sensors. Additionally, we reveal that *CcPKCα* phosphorylation and AKH/AKHR signaling act as downstream mediators of TRPV heteromeric tetramers. Crucially, we demonstrate evolutionary conservation of this CcIav/CcNan‐CcPKCα‐AKH/CcAKHR axis across three insect orders. Our findings not only resolve the thermosensory logic of seasonal polyphenism of *C. chinensis* but also pioneer receptor‐targeted strategies to disrupt psyllid population cycles.

## 2 Results

### 2.1 Experimental Model for 25 °C Temperature Triggering the Seasonal Transition of Winter‐ to Summer‐Form in *C. chinensis*

**Figure 1:**
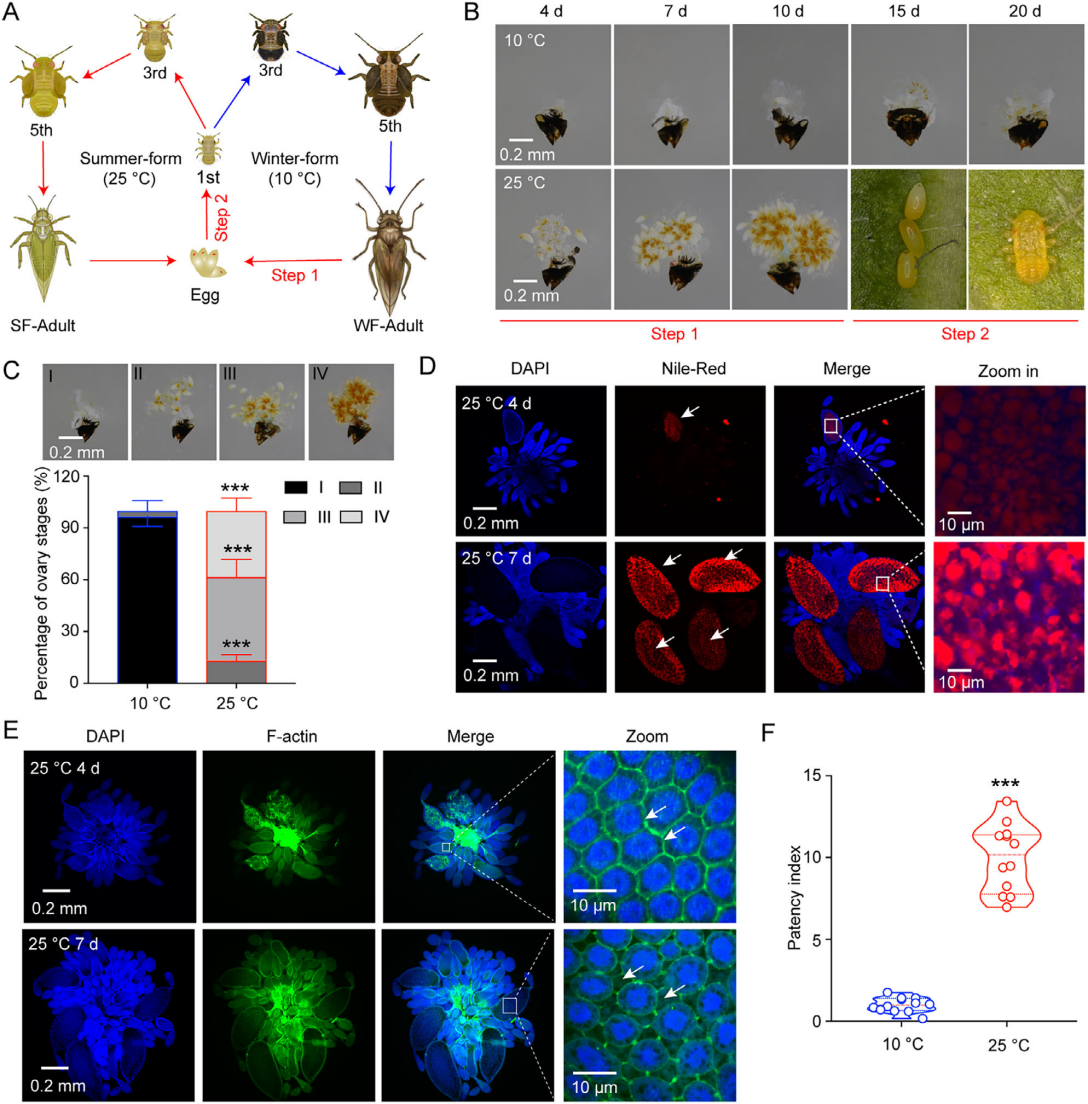
At 25 °C, temperature‐induced winter‐ to summer‐form transition through lipid metabolism and ovarian remodeling in *C. chinensis*. A) Schematic of temperature‐dependent morph transition: Summer‐form (SF, red arrows) predominates at 25 °C, while winter‐form (WF, blue arrows) persists at 10 °C. B) Ovarian development in newly emerged WF females at 10 °C versus 25 °C (d: days post‐emergence). Scale: 0.2 mm. C: Stage distribution of WF ovaries at 8 d post‐emergence at 10 and 25 °C conditions (*n* = 3 replicates; ≥ 30 insects/replicate). Stages: I (1–3 d), II (4–6 d), III (7–9 d), and IV (10–12 d). D,E) Thermal regulation of lipid droplets (Nile Red) and follicular patency index (phalloidin/DAPI) in newly emerged WF females. Scale bar in 1D and 1E: 0.2 mm. Nuclei (blue, DAPI). Lipid droplets (Nile red, red; arrowhead). F‐actin in the follicular epithelium (phalloidin, green; arrowhead). F) Quantification of follicular patency index (mean ± SEM; *n* = 12 technical replicates). Data in 1C and 1F: Mean ± SEM from 3 independent biological replicates, with each replicate comprising at least 30 insects. Statistical significance (^***^ *p* < 0.001) between groups was determined using the pair‐wise Student's *t*‐test in Graphpad Prism 8.0 software.

Previous studies have established that *C. chinensis* exhibits distinct summer‐ and winter‐form under 25 °C and 10 °C conditions, respectively.^[^ ^2^ ^]^ Our experimental model reveals that the winter‐to‐summer form transition involves two critical phases: i) 25 °C exposure terminates reproductive diapause in winter‐form females, triggering rapid ovarian development and oviposition; and ii) subsequent embryonic development yields summer‐form nymphs (**Figure** **1**A). Comparative analysis demonstrated striking temperature‐dependent differences in ovarian development. At 25 °C, winter‐form adults exhibited accelerated ovarian maturation and successful oviposition, ultimately transforming into summer‐form nymphs. In contrast, ovaries remained developmentally arrested at 10 °C (Figure 1B). Using our established four‐stage classification system,^[^ ^10^ ^]^ we observed significantly higher proportions of Stage II–IV ovaries in 25 °C‐treated winter‐form females by day 8 post‐emergence compared to 10 °C controls (Figure 1C). Thermal stimulation at 25 °C consistently upregulated multiple physiological markers: Energy reserves (Triglycerides and glycogen), Lipid droplet accumulation, Vitellogenesis genes (*CcVg1*, *CcVg2*, *CcVgR*), Follicular epithelium patency index (Figure 1D–F; Figure S1, Supporting Information). These findings support a model wherein 25 °C‐induced transition from winter‐ to summer‐form is mediated through coordinated follicular epithelium remodeling (enhanced patency), nutrient mobilization (lipid/glycogen accumulation), vitellogenin production and uptake.

### 2.2 Molecular Characterization of *CcIav*/*CcNan* Heterotetramers as Thermosensors in *C. chinensis*

**Figure 2:**
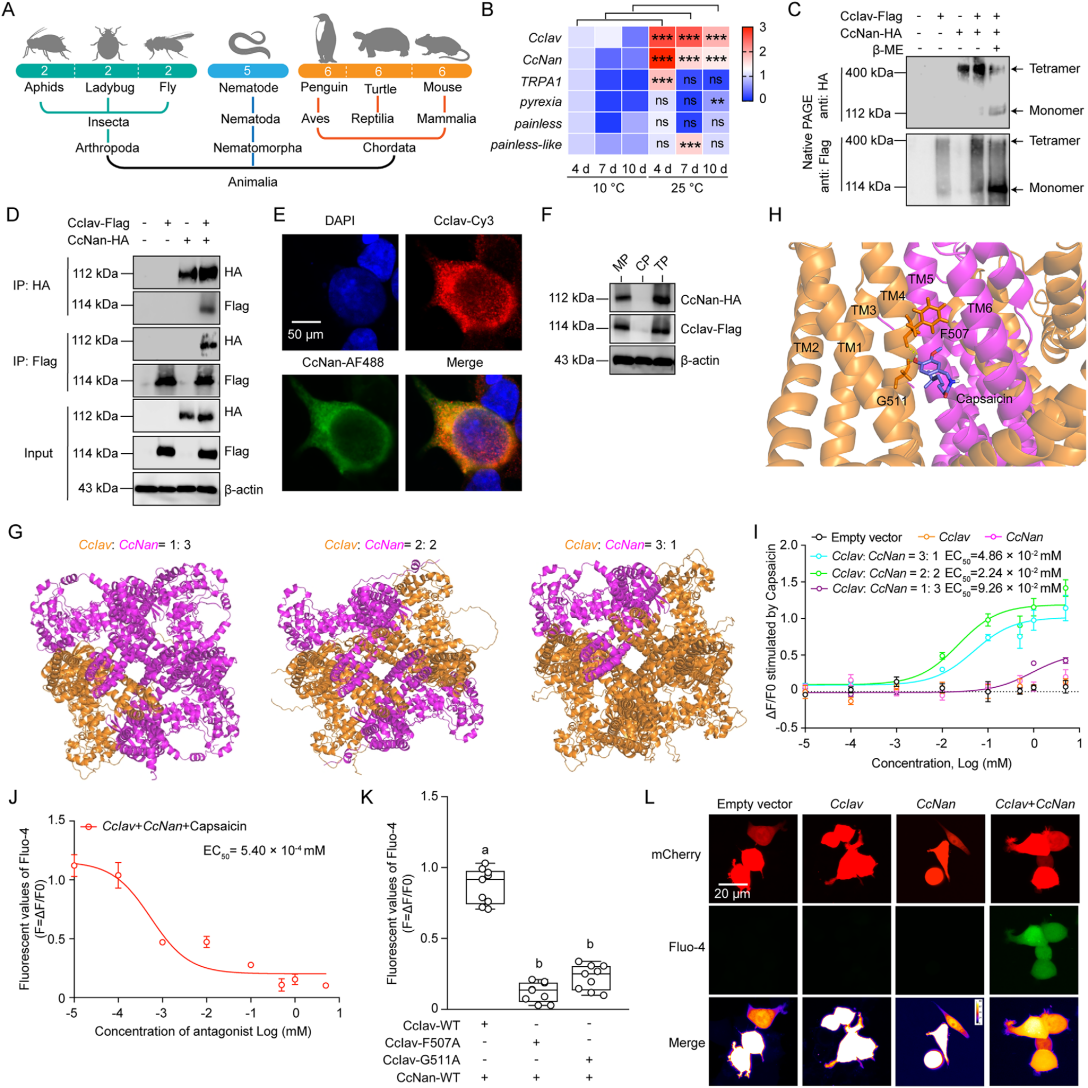
Thermosensory function of TRPV heterotetramer CcIav/CcNan in *C. chinensis*. A) Phylogenetic tree analysis of TRPV subunits across seven species. B) Temperature‐dependent expression dynamics of TRPV/TRPA channel subunits (*n* = 3 biological × 3 technical replicates). C) Native‐PAGE analysis of CcIav/CcNan oligomerization. β‐Mercaptoethanol (β‐ME) disrupts disulfide bonds. D) Co‐immunoprecipitation (Co‐IP) of Flag‐CcIav and HA‐CcNan in HEK293T cells. E) Confocal imaging showing colocalization of CcIav‐Cy3 (red) and CcNan‐AF488 (green) in HEK293T cells. Nuclei (blue, DAPI). F) Subcellular fractionation: membrane (MP) versus cytoplasmic (CP) localization of Flag‐CcIav and HA‐CcNan. G) AlphaFold3‐predicted configurations of CcIav/CcNan heterotetramers at different molar ratios (1:3, 2:2, 3:1). H) Capsaicin (yellow) docking at CcIav‐CcNan (2:2) interface (Key residues: F507, G511of CcIav). I) Capsaicin dose‐response curves in HEK293T cells expressing CcIav/CcNan (EC50 indicated; empty vector control). J) Inhibition of capsaicin‐induced calcium flux by TRPV antagonist SB‐366791. K) Reduced capsaicin sensitivity in HEK293T cells expressing CcIav mutants (F507A, G511A) with CcNan. L) Ca^2+^ imaging (Fluo‐4, green) in HEK293T cells expressing *CcIav*, *CcNan*, or both to capsaicin treatments (mCherry vector control). The negative control involved HEK293T cells expressing an empty plasmid (pcDNA3.1(+)‐mCherry) treated with capsaicin. The red and green colors represented mCherry and Fluo‐4 signals, respectively. Merge: merged imaging of mCherry and Fluo‐4 signals. Scale bar: 20 µm. Data in (I–K) Mean ± SEM (*n* = 6 for I and J; *n* = 9 for K); Statistical significance (^***^ *p* < 0.001) between groups was determined using the pair‐wise Student's *t*‐test in Graphpad Prism 8.0 software. Letters denote significance (ANOVA, Tukey's HSD; *p* < 0.05).

Comparative genomic analysis revealed insects uniquely retain only two TRPV subunits (Iav and Nan), contrasting with six in chordates and five in nematodes (**Figure** **2**A). In *C. chinensis*, *CcIav* and *CcNan* showed significant upregulation at 25 °C versus 10 °C, while TRPA channels remained unresponsive (Figure 2B). Both subunits exhibited >80% identity in transmembrane domains with other orthologs (Figures S2 and S3, Supporting Information).

Native PAGE demonstrated CcIav forms monomeric/homotetrameric, while CcNan primarily assembles as homotetramers (β‐Mercaptoethanol‐sensitive). Co‐expressed subunits formed stable heterotetramers (Figure 2C), confirmed by: i) Co‐IP assays demonstrating physical interaction between CcIav‐Flag and CcNan‐HA (Figure 2D); ii) Membrane co‐localization (Figure 2E); iii) Cellular fractionation and Western blot results confirming predominant membrane distribution (Figure 2F). Pharmacological characterization identified that capsaicin is a dose‐dependent activator and SB‐366791 is a dose‐dependent antagonist for *CcIav* and *CcNan* (Figure S5A–D, Supporting Information). Alphafold3 predicted three heterotetrameric configuration of CcIav and CcNan (1:3: ipTM = 0.63, pTM = 0.64; 2:2: ipTM = 0.67, pTM = 0.67; and 3:1: ipTM = 0.64, pTM = 0.66), with the 2:2 complex showing highest stability (Figure 2G; Figure S4A‐C, Supporting Information). Molecular docking unveiled a capsaicin binding pocket between CcIav (TM3‐TM4) and CcNan (TM5‐TM6) and involving key residues G507/F511 (–6.27 kcal mol^−1^) (Figure 2H), resembling the interaction observed in human TRPV1.^[^ ^32^, ^33^ ^]^ The 2:2 heterotetramer exhibited greatest capsaicin sensitivity (EC_50_ value of 2.24 × 10^−5^ mm; Figure 2I), reversibly inhibited by TRPV antagonist SB‐366791^[^ ^34^ ^]^ (Figure 2J). Mutating F507/G511 abolished activation (Figure 2K; Figure S4D,E, Supporting Information), while calcium imaging confirmed functional coupling to Ca^2+^ signaling (Figure 2L). In conclusion, CcIav and CcNan are warm temperature‐sensitive ion channels activated by 25 °C and capsaicin in *C. chinensis*.

### 2.3 Essential Role of CcIav/CcNan Heterotetramers in the Winter‐to‐Summer‐Form Transition

**Figure 3:**
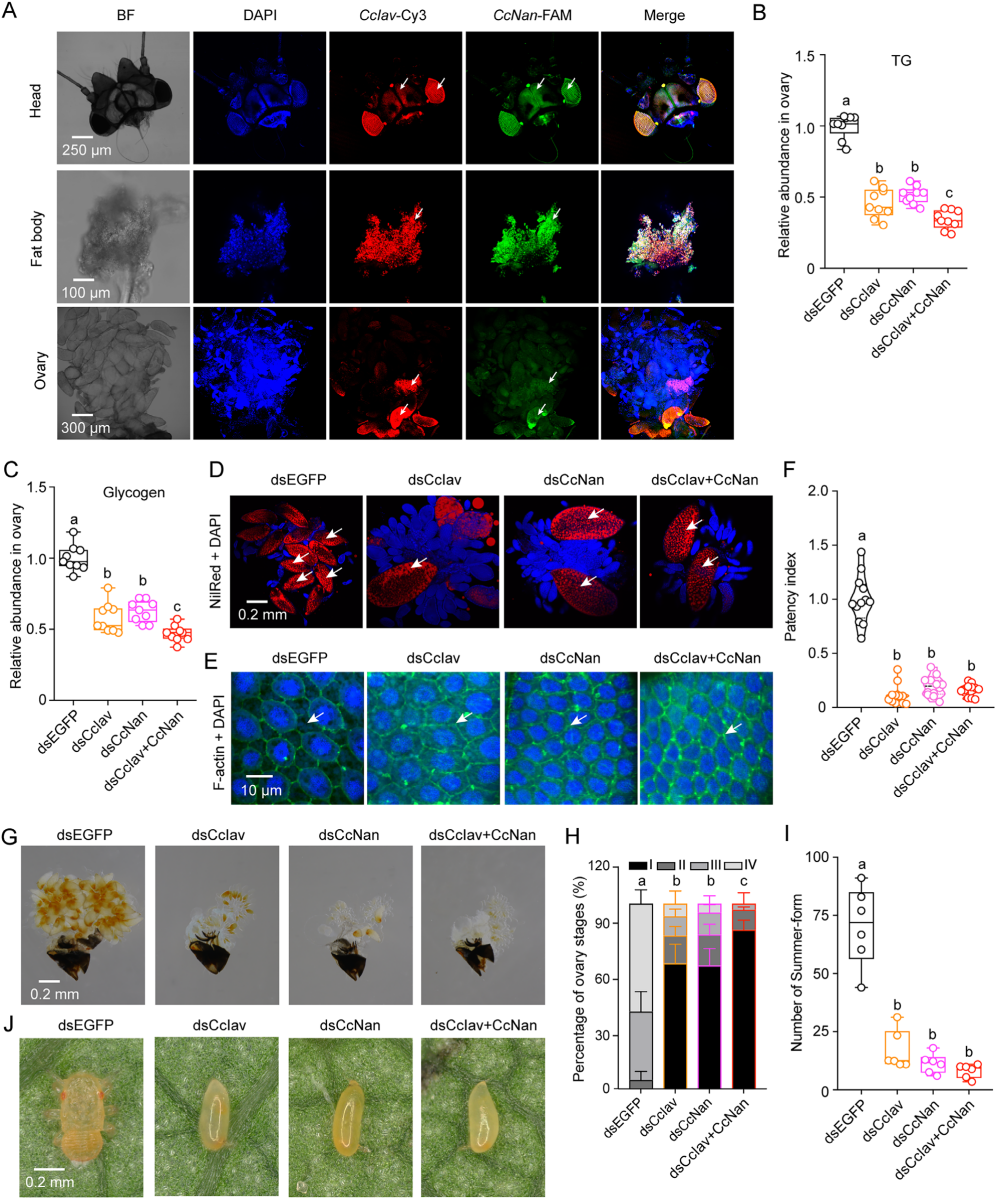
Essential role of CcIav/CcNan heterotetramers in orchestrating winter‐to‐summer‐form transition. A) Tissue‐specific co‐localization of *CcIav* (Cy3, red) and *CcNan* (FAM, green) mRNA by FISH. Nuclei (DAPI, blue). BF: bright field. Scale bars: Head (250 µm), Fat body (100 µm), Ovary (300 µm). B,C) Effects of dsRNA‐mediated knockdown (dsCcIav, dsCcNan, or combined) on whole‐body triglyceride (B) and glycogen (C) levels in WF females. D,E) Effects of RNAi on ovarian lipid droplets (Nile Red, red; D) and follicular patency (phalloidin, green; E) in WF females. Scale bars: 0.2 mm (D), 10 µm (E). F) Quantification of follicular patency index after RNAi. G–I) Phenotypic consequences of RNAi on ovary development (G), morph transition rate, and representative adult morphs (I) at 5 DAE under 25 °C. Scale bar: 0.2 mm (G). J) Representative winter‐form eggs laid by WF females after RNAi. Data are presented as mean ± SEM (*n* = 9 in B and C; *n* = 3 in H, ≥30 insects/replicate; *n* = 6 in I). Letters denote significance (ANOVA, Tukey's HSD; *p* < 0.05).

Tissue‐specific expression profiling unveiled widespread expression of *CcIav* and *CcNan*, with particularly heightened levels in head and fat body (Figure S6A,B, Supporting Information). FISH confirmed their co‐expression in the head, fat body, and ovaries (**Figure** **3**A). RNAi‐mediated phenotypic effects on winter‐form females (5 days after eclosion, DAE) under 25 °C showed effective target knockdown of *CcIav* (Figure S6C, Supporting Information) and *CcNan* (Figure S6D, Supporting Information); tissue‐specific knockdown efficiency of *CcIav* and *CcNan* in head, fat body, and ovary (Figure S6E,F, Supporting Information). Metabolic assessments revealed that RNAi‐mediated suppression of *CcIav* and/or *CcNan* led to a significant decrease in triglyceride levels, reduced glycogen content, and diminished lipid droplet determination (Figure 3B‐D). qRT‐PCR data further indicated that knockdown of *CcIav*, *CcNan*, or both led to a marked inhibition in the mRNA expression of *CcVg1*, *CcVg2*, and *CcVgR* (Figure S6G–I, Supporting Information). Morphological assessments demonstrated substantial impacts on: follicular epithelium patency, patency index, ovary development, and transition from winter‐ to summer‐form in winter‐form females at 5 DAE (Figure 3E‐I). Pharmacological interventions showed: TRPV antagonist mimicked RNAi effects, while capsaicin only partially rescued the transition phenotype (Figure S6J, Supporting Information). In order to ascertain that the influence of *CcIav* and *CcNan* is not limited solely to the ovaries, we assessed their impact on the transition of eggs laid by winter‐form female adults under 25 °C conditions. After successful knockdown of *CcIav*, *CcNan*, or both, a noticeable decrease in the percent of eggs transitioning to summer‐form nymphs was observed compared to dsEGFP treatments (Figure 3J; Figure S7A–C, Supporting Information). Control experiments using summer‐form adults at 25 °C showed no significant effects on: vitellogenesis gene expression (*CcVg1*, *CcVg2*, and *CcVgR*), ovarian development, and egg production (Figure S8A–F, Supporting Information).

### 2.4 CcPKCα Phosphorylation as a Key Downstream Signal of the Receptors CcIav and CcNan in Regulating Seasonal Polyphenism of *C. chinensis*

**Figure 4:**
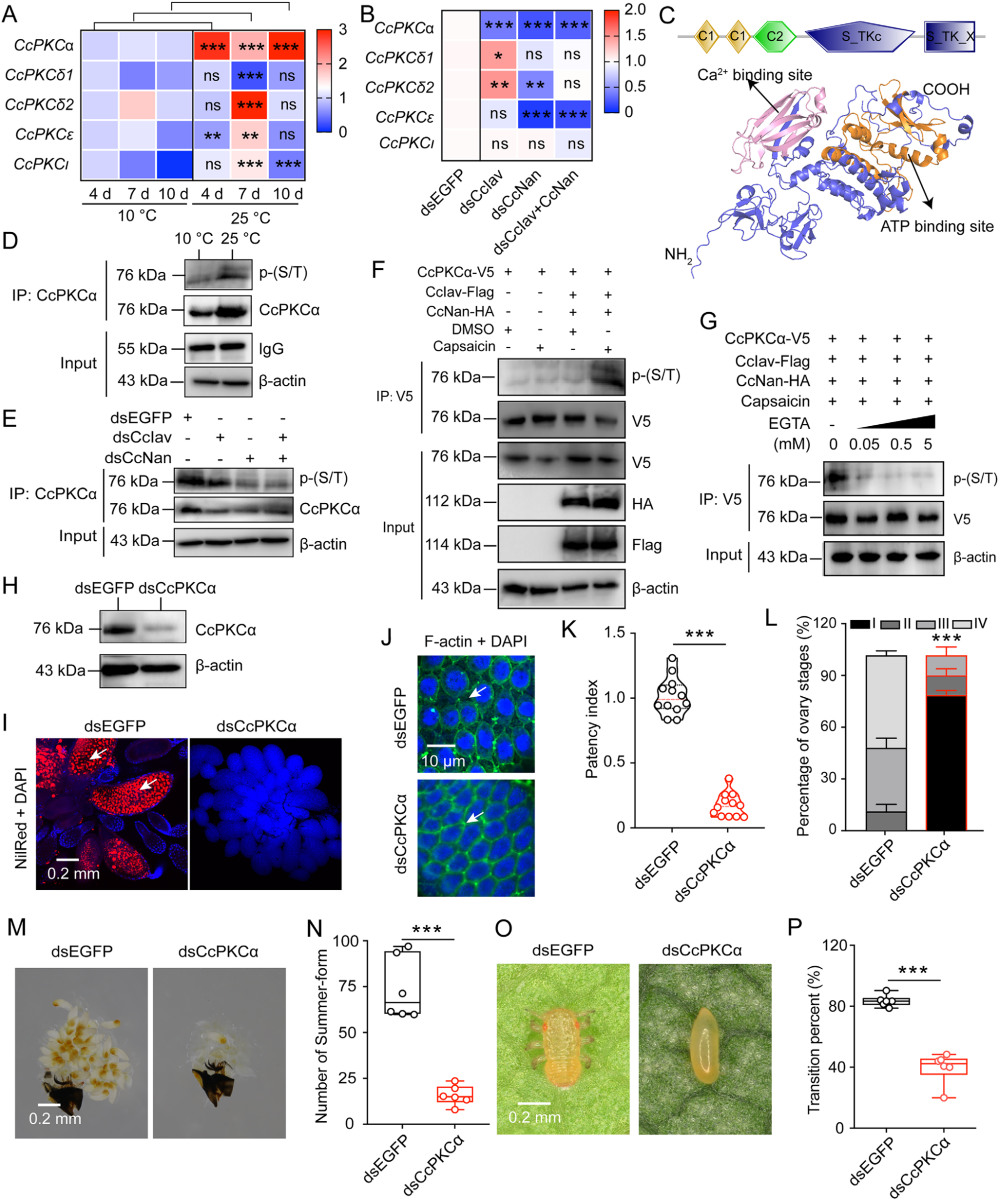
CcPKCα phosphorylation mediates *CcIav*/*CcNan*‐dependent seasonal polyphenism in *C. chinensis*. A) Heatmap of PKC isoform expression in WF females at 10 °C versus 25 °C. B) Effect of *CcIav*/*CcNan* knockdown on PKC transcripts. C) AlphaFold3‐predicted CcPKCα structure highlighting conserved domains (C1, C2, and S_TKc). Arrowheads denote Ca^2+^/ATP binding sites. D,E) WB analysis of CcPKCα protein (D) and phosphorylation (p‐CcPKCα; E) in WF females under thermal or RNAi treatments. F,G) Capsaicin activation (F) and EGTA suppression (G) of p‐CcPKCα in HEK293T cells expressing CcIav/CcNan. H) WB validation of dsCcPKCα knockdown efficiency. I,J) Effects of CcPKCα knockdown on ovarian lipid droplets (Nile Red, red; I) and follicular patency (phalloidin, green; J). Scale bars: 0.2 mm (I), 10 µm (J). K) Quantification of follicular patency index after dsCcPKCα. L) Ovarian stage distribution post dsCcPKCα treatment. M,N) Effects of dsCcPKCα on ovary development (M) and morph transition rate (N). Scale bar: 0.2 mm (M). O,P) Phenotype of WF eggs post RNAi of *CcPKCα*. Scale: 0.2 mm (I, M, and O); 10 µm (J). Data are presented as Mean ± SEM (*n* = 3 in A, B, and L; *n* = 6 in N and P; ≥30 insects per replicate). Significance: ^**^ *p* < 0.01, ^***^ *p* < 0.001 (Student's *t*‐test); n.s.: no significant (*p* > 0.05).

Consistent with established TRP channel signaling mechanisms,^[^ ^21^ ^]^ we investigated the Ca^2+^‐mediated protein kinase C pathway downstream of CcIav‐CcNan heterotetramers. Temperature‐dependent qRT‐PCR revealed selective upregulation of *CcPKCα* (but not other isoforms) at 25 °C versus 10 °C (**Figure** **4**A). Knockdown of *CcIav*/*CcNan* similarly decreased *CcPKCα* mRNA expression (Figure 4B), establishing its position downstream of TRPV activation. *CcPKCα* (669 aa, 76.55 kDa) contains three conserved region domains (two C1 and one C2) alongside two Serine/Threonine protein kinases catalytic domains (Figure 4C). Utilizing Alphafold3 and PyMOL‐v1.3r1 software, we constructed the potential tertiary protein structure of CcPKCα, identifying conserved Ca^2+^ and Adenosine triphosphate (ATP) binding sites (Figure 4C). Multiple alignment analysis highlighted significant conservation in the functional domains of CcPKCα when compared with PKCα sequences from six other selected insect species (Figure S9, Supporting Information). Phylogenetic analysis revealed closest homology to DcPKCα (*D. citri*, XP_02 668 5144.1) (Figure S10, Supporting Information), with the highest expression in the head (Figure S11A, Supporting Information).

Co‐IP/Western blot demonstrated that: i) increased CcPKCα phosphorylation at 25 °C (Figure 4D); ii) reduced protein expression and phosphorylation upon *CcIav*/*CcNan* knockdown (Figure 4E); iii) calcium agonist Ionomycin notably elevated *CcPKCα* expression, while the calcium chelating agent EGTA inhibited *CcPKCα* mRNA level (Figure S11B,C, Supporting Information); iv) capsaicin‐induced phosphorylation via CcIav/CcNan, whereas EGTA‐mediated suppression of phosphorylation (Figure 4F,G). These data collectively suggest that *CcPKCα* functions as a pivotal downstream signal of the receptors *CcIav* and *CcNan*.

To delve into the role of *CcPKCα* in the transition from winter‐ to summer‐form in *C. chinensis*, we conducted RNAi‐mediated knockdown of *CcPKCα* expression. Successful knockdown of *CcPKCα* substantially impacted triglyceride levels, glycogen content, lipid droplets, mRNA expression of *CcVg1*, *CcVg2*, and *CcVgR*, follicular epithelium patency, patency index, ovary development, and transition from winter‐ to summer‐form in winter‐form females at 5 DAE (Figure 4H‐N; Figure S11D,G, Supporting Information). When knocking down *CcPKCα* in eggs, the transition from winter‐ to summer‐form was also blocked (Figure 4O,P). Taken together, phosphorylation of CcPKCα emerges as a crucial downstream signal mediating the regulatory effects of CcIav and CcNan on the seasonal polyphenism of *C. chinensis*.

### 2.5 *CcIav*/*CcNan* Heterotetramers Regulate *CcAKHR* Phosphorylation via *CcPKCα*

Given the established role of AKH signaling in energy metabolism and ovarian development, we investigated its involvement in the seasonal transition from winter to summer in the following five aspects.

**Figure 5:**
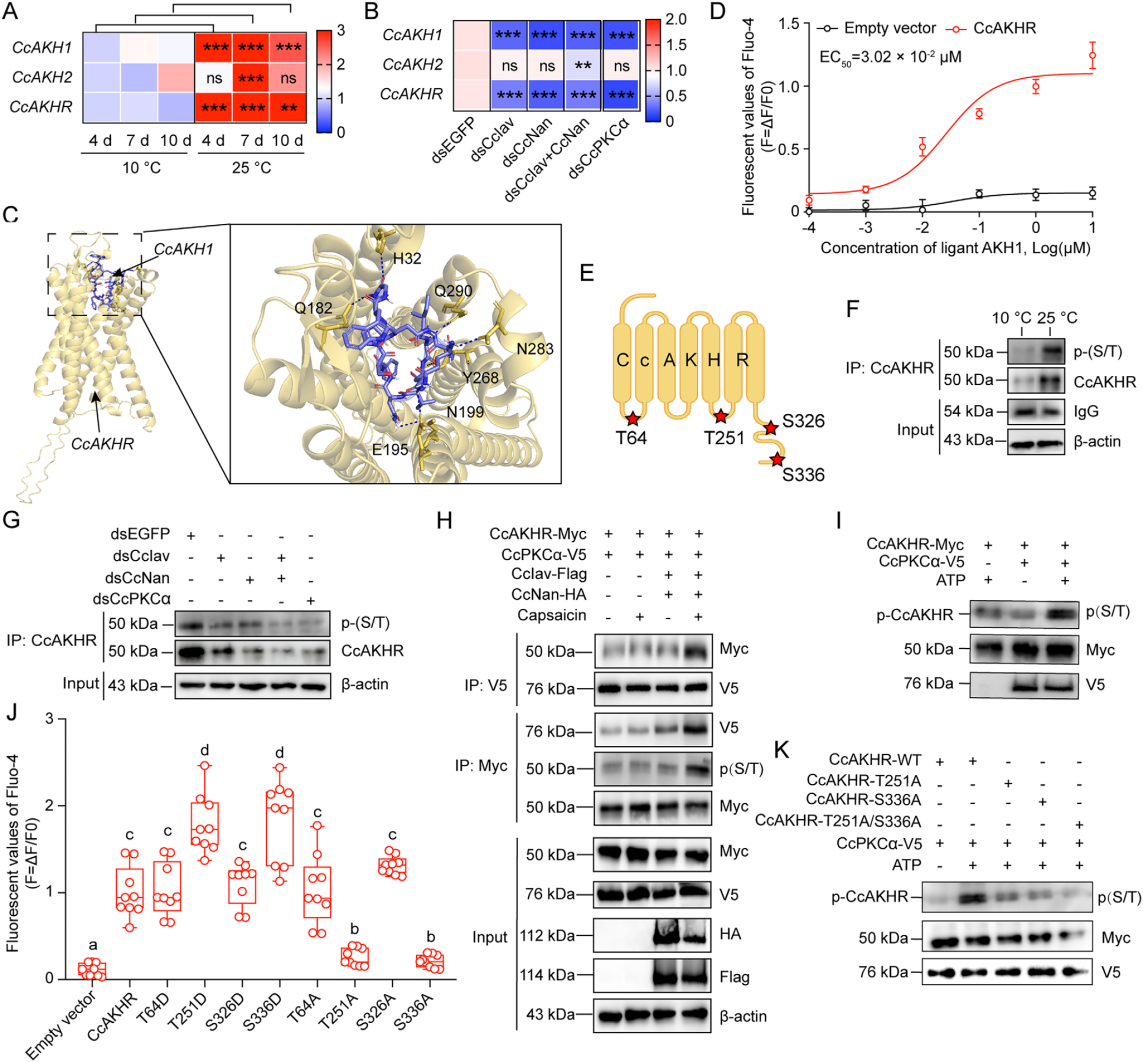
CcIav/CcNan triggers CcAKHR phosphorylation through CcPKCα. A) Thermal regulation of *CcAKH* and *CcAKHR* expression. B) Effect of *CcIav*, *CcNan*, or *CcPKCα* knockdown on *CcAKH*/*CcAKHR* transcript levels. C) Predicted structure of CcAKHR and its interaction interface with CcAKH1 peptide. D) CcAKH1‐induced calcium flux (Fluo‐4 imaging) in CcAKHR‐expressing HEK293T cells. E) Predicted intracellular phosphorylation sites (Thr/Ser) on CcAKHR. F,G) Regulation of CcAKHR phosphorylation (p‐CcAKHR) by temperature (F) and CcIav, CcNan, or CcPKCα (G) in vivo. H) Capsaicin activation of p‐CcAKHR in HEK293T cells co‐expressing CcIav/CcNan, CcPKCα, and CcAKHR. I–K) In vitro phosphorylation of CcAKHR by CcPKCα and mutation validation. Data are presented as Mean ± SEM (*n* = 3 in A, B, ≥30 insects/replicate; *n* = 6 for D; and *n* = 9 for J). Significance: ^**^ *p* < 0.01, ^***^ *p* < 0.001 (Student's *t*‐test); n.s.: *p* > 0.05; Letters denote significance (ANOVA, Tukey's HSD; *p* < 0.05).

1. **Temperature‐Dependent Regulation**: qRT‐PCR revealed significant upregulation of *CcAKH1* and *CcAKHR* mRNA levels at 25 °C versus 10 °C; Knockdown of *CcIav*, *CcNan*, a combination of both, or *CcPKCα* substantially reduced their expression levels, while *CcAKH2* remained unaffected (**Figure** **5**A,B).
2. **Molecular Characterization**: *CcAKH1* encodes a 72 aa residues with mature peptide “QVNFSPNWGG” exhibited significant homology with counterparts from other selected insect species (Figure S12A, Supporting Information); CcAKHR transmembrane domains showed high conservation across species (Figure S13, Supporting Information); Phylogenetic assessments positioned closest homology of CcAKH1 to its DcAKH1 counterpart (*D. citri*, XP_0 084 88257.1), while CcAKHR with DcAKHR ortholog (*D. citri*, AWT50656.1), underlining their significance as Hemiptera pest of fruit trees (Figures S12 and S13, Supporting Information).
3. **Functional Interactions**: Molecular docking identified seven CcAKH1 binding sites (Gln182, His32, Gln290, Asn283, Tyr268, Asn199, and Glu195) on CcAKHR (Figure 5C); Calcium mobilization assays demonstrated a dose‐dependent activation of CcAKHR by CcAKH1 (EC_50_ = 3.02 × 10^−2^ µM), while no response was elicited with an empty vector (Figure 5D); Four phosphorylation sites (Threonine 64 and 251, Serine 326 and 336) identified in CcAKHR (Figure 5E).
4. **Thermal and Genetic Modulation**: At 25 °C increased CcAKHR protein and phosphorylated protein levels versus 10 °C (Figure 5F); *CcIav*/*CcNan*/*CcPKCα* knockdown reduced both protein expression and phosphorylated protein levels of CcAKHR (Figure 5G); Capsaicin enhanced CcAKHR protein and phosphorylation via CcIav/CcNan/CcPKCα; Exogenous ATP treatment boosted these levels via CcPKCα (Figure 5H,I).
5. **Phosphorylation‐Dependent Activation**: Mutational analyses further elucidated that mimicking phosphorylation at threonine 251 and serine 336 potentiated the activation of CcAKH1 on CcAKHR, while mimicking non‐phosphorylated forms reduced this activation (Figure 5J). Co‐IP and Western blot analyses revealed a significant decrease in both the protein expression and phosphorylated protein levels of CcAKHR when mutating threonine at position 251, serine at position 336 to aspartic acid, or both, in response to exogenous ATP treatment (Figure 5K). These results collectively suggest that heterotetramers of CcIav and CcNan induce CcAKHR phosphorylation through CcPKCα, highlighting a complex regulatory network governing seasonal transitions in *C. chinensis*.

### 2.6 *CcAKH1* Engagement with Its Receptor *CcAKHR* Orchestrates the Winter‐ to Summer‐Form Transition in *C. chinensis*

**Figure 6:**
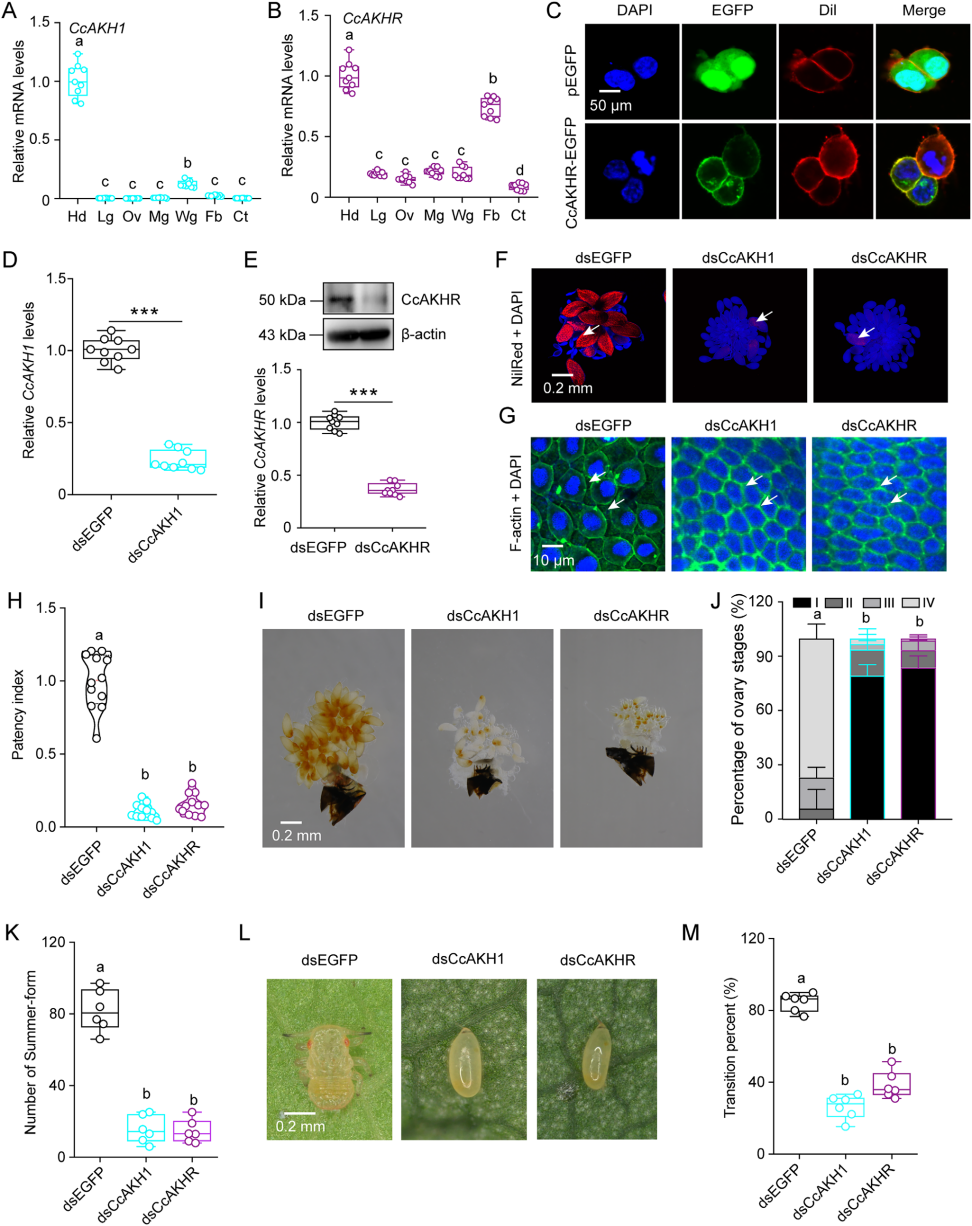
*CcAKH1*/*CcAKHR* signaling drives winter‐to‐summer‐form transition in *C. chinensis*. A,B) Tissue‐specific expression patterns of *CcAKH1* and *CcAKHR* via qRT‐PCR. Hd: head, Lg: leg, Ov: ovary, Mg: midgut, Wg: wing, Fb: fat body, Ct: cuticle. C) Subcellular localization of CcAKHR‐EGFP (green) in HEK293T cells. Membrane: Dil (red); nuclei: DAPI (blue). Scale bar: 50 µm. D,E) Validation of *CcAKH1* and *CcAKHR* knockdown efficiency by qRT‐PCR or WB. F–K) Phenotypic consequences of dsCcAKH1/dsCcAKHR treatments on lipid droplet depletion (Nile Red), follicular patency defects (phalloidin), patency index quantification, ovary development arrest, and morph transition. L–M) Phenotype of WF eggs after RNAi of dsCcAKH1/dsCcAKHR. Scale bars: 50 µm (C); 0.2 mm (F, I, and L); 10 µm (G). Data are presented as mean ± SEM (*n* = 3 for A, B, D‐E, and J; *n* = 6 for K and M; ≥30 insects per replicate). Significance: ^***^ *p* < 0.001 (Student's *t*‐test); letters denote ANOVA/Turkey's HSD (*p* < 0.05). Letters denote significance (ANOVA, Tukey's HSD; *p* < 0.05).

qRT‐PCR analysis revealed predominant expression of both *CcAKH1* and *CcAKHR* in the head region compared to six other examined tissues (**Figure** **6**A,B). Subcellular localization studies pinpointed membrane‐specific distribution of *CcAKHR* (Figure 6C). To delve deeper into the role of AKH signaling in the seasonal transition from winter‐ to summer‐form in *C. chinensis*, RNAi assays were performed. Treatment with dsCcAKH1 led to a significant 76% reduction in *CcAKH1* mRNA levels, while exposure to dsCcAKHR resulted in a noticeable decrease in both mRNA and protein levels of CcAKHR, in stark contrast to dsEGFP treatments (Figure 6D,E). Effective knockdown of *CcAKH1* and *CcAKHR* resulted in impaired energy metabolism (reduced triglyceride levels, decreased glycogen content, diminished lipid droplet accumulation), disrupted vitellogenesis (downregulated *CcVg1*, *CcVg2*, and *CcVgR* expression), ovarian developmental defects (impaired follicular epithelium patency, reduced patency index, arrested ovary development), and blocked winter‐ to summer‐form transition (Figure 6F–M; Figure S14A–E, Supporting Information). Collectively, these findings underscore the indispensable role of AKH signaling, encompassing *CcAKH1* and *CcAKHR*, in orchestrating the seasonal polyphenism observed in *C. chinensis*.

### 2.7 A Conserved Mechanism of Inactive and Nanchung Forming Heterotetramers to Perceive 25 °C Temperature and Capsaicin Across Various Insect Species

To elucidate the conservation of the mechanism by which two TRPV subunits sense 25 °C across species, we focused on three key orchard insects: *D. citri*, *H. axyridis*, and *B. dorsalis*. Our temperature treatment experiments revealed a significant upregulation of mRNA expression for six TRPV subunits, *DcIav*, *DcNan*, *BdIav*, *BdNan*, *HaIav*, and *HaNan*, after 25 °C exposure for 4, 7, and 10 days compared to the 10 °C control (Figure S15, Supporting Information). Utilizing Alphafold3 predictions, we observed the formation of heterotetramers composed of Iav and Nan subunits in the selected insect species at a 2:2 ratio (Figure S16A–C, Supporting Information). Molecular docking analyses unveiled a capsaicin binding pocket between the transmembrane helices TM3‐TM4 of Iav and TM5‐TM6 of CcNan, mirroring interactions seen in *C. chinensis* and humans (Figure S16D–F, Supporting Information). Furthermore, as depicted in Figure S16G–I (Supporting Information), the Iav‐Nan heterotetramers exhibited dose‐dependent activation by capsaicin, with EC_50_ values of 5.78 × 10^−2^ mm, 2.41 × 10^−2^ mm, and 2.45 × 10^−2^ mm at a 2:2 molar ratio in *D. citri*, *H. axyridis*, and *B. dorsalis*, respectively. Validation through Co‐IP assays using anti‐HA and anti‐Flag antibodies confirmed the co‐immunoprecipitation of Iav‐Flag with Nan‐HA when co‐expressed in HEK293T cells across the three species (Figure S16J–L, Supporting Information). These data suggest that the mechanism by which Inactive and Nanchung form heterotetramers to sense 25 °C temperature and capsaicin is evolutionarily conserved among diverse insect species.

At 10 °C, the CcIav/CcNan‐CcPKCα‐AKH/CcAKHR axis remains inactive (gray arrows), maintaining winter‐form ovarian stasis and blocking transition. Conversely, at 25 °C, thermal activation triggers: i) CcIav/CcNan heterotetramers assembly → calcium influx across the membrane receptor; ii) CcPKCα phosphorylation → Enhanced and activated AKH signaling; iii) CcAKH1 binding to membrane‐localized CcAKHR → Mobilization of energy stores (lipid droplet depletion, glycogenolysis) and induction of follicular patency for vitellogenin transport; iv) Oocyte maturation → Summer‐form nymph production and population expansion. Red arrows: Activated pathways; Gray arrows: Inactivated states.

## 3 Discussion

Seasonal polyphenism, a widespread phenomenon across diverse taxa, fundamentally enhances organismal fitness by enabling rapid adaptation to environmental fluctuations. This adaptive strategy optimizes survival and reproduction success through various environmental cues, including temperature, photoperiod, and nutritional availability.^[^ ^35^ ^]^ Although temperature represents a paramount driver of seasonal polyphenism, the mechanistic basis and evolutionary dynamics underlying thermal regulation remain inadequately understood. Recent advances utilizing *C. chinensis* as a model organism have pinpointed *CcTRPM* and *CcTRPC3* as cold‐sensitive receptors orchestrating the summer‐ to winter‐form transitions.^[^ ^2^, ^10^ ^]^ However, the mechanisms underlying warm temperature perception and the subsequent shift from winter‐ to summer‐form in *C. chinensis* remain enigmatic. Notably, winter‐form adults maintained at 10 °C show minimal ovarian changes, whereas exposure to 25 °C triggers rapid ovarian development and transition to summer‐form nymphs (Figure 1A,B). Our experimental results successfully established a robust laboratory model demonstrating that 25 °C treatment induces winter‐ to summer‐form transition, characterized by enhanced ovarian development, heightened energy metabolites accumulation, upregulated vitellogenin mRNA expression, and elevated follicular epithelium permeability in winter‐form females (Figure 1C–F). These findings clearly underscore that the winter‐ to summer‐form transition primarily involves ovarian development changes, contrasting with the epidermal compositional alterations that characterize the summer‐ to winter‐form transition in *C. chinensis*.

To identify the molecular sensor mediating 25 °C perception, we systematically analyzed TRPV and TRPA homologs, known thermal sensors in both humans and insects. qRT‐PCR results revealed a substantial upregulation in mRNA levels of *CcIav* and *CcNan* following 25 °C exposure and capsaicin treatments. Complementary protein interaction assays and cellular studies confirmed that CcIav‐CcNan heterotetramers function as molecular sensors for these thermal/chemical stimuli (Figure 2). These observations align with established principles of TRP channel biology, where tetrameric assemblies (homomeric or heteromeric) govern calcium influx.^[^ ^14^ ^]^ Comparative structural analyses reinforce this mechanism: mammalian TRPV1/TRPV5 form homotetramers with cryo‐EM‐resolved architectures resembling voltage‐gated ion channels,^[^ ^36^, ^37^ ^]^ while *Caenorhabditis elegans* TRPV subunits OSM‐9 and OCR‐2 assemble into heteromers as demonstrated by total internal reflection (TIRF) microscopy photobleaching assays.^[^ ^38^ ^]^ Additional examples of cross‐subfamily assemblies include TRPC1‐TRPV4 complexes^[^ ^39^ ^]^ and ternary complexes containing TRPV4, TRPC1, and TRPP2,^[^ ^15^ ^]^ underscoring the structural and functional diversity conferred by heteromeric co‐assembly. Beyond *C. chinensis*, conserved heterotetrameric TRPV functionality in *D. citri*, *H. axyridis*, and *B. dorsalis* indicates its broad role in insect thermosensing (Figure S16, Supporting Information). Moreover, phylogenetic divergence between insect and mammalian TRPVs (Figure S3, Supporting Information), combined with domain‐specific sequence variations across insect species (Figure S2, Supporting Information), provides a molecular basis for targeted insecticide design. Building on TRPV's function as a pymetrozine target, our findings support developing precision insecticides that disrupt TRPV thermosensing modules—exploiting evolutionary divergence to compromise pest thermal adaptation while minimizing off‐target risks to mammals, beneficial insects, and pollinators.

Given the pivotal role of CcIav‐CcNan heterotetramers in initiating calcium‐permeable channel receptor regulating seasonal polyphenism, we investigated the downstream PKC signal cascade. Our transcriptional and protein levels analyses unveiled that CcIav‐CcNan heterotetramers stimulate both expression and phosphorylation of CcPKCα (Figure 4A‐G). We found that CcPKCα modulates the transition from winter‐ to summer‐form by enhancing energy metabolism and ovarian development in winter‐form females (Figure 4H‐P). These findings parallel observations in mammal systems, where TRPV1 activation by lipoxygenase metabolites requires PKC phosphorylation in trigeminal ganglion neurons,^[^ ^21^ ^]^ and where PKCβII regulates the basal thermal sensitivity of TRPV1 ion channels in mouse sensory neurons.^[^ ^40^ ^]^ Given the substantial energy requirement associated with the transition from winter‐ to summer‐form, we subsequently delved into investigating the function of endocrine hormone signaling.

Recent studies have highlighted the pivotal role of the AKH/membrane receptor AKHR signaling pathway in promoting yolk production and nutrient mobilization during offspring rearing.^[^ ^24^, ^29^, ^41^ ^]^ However, its involvement in seasonal polymorphism remains unexplored. Our investigations address this knowledge gap by demonstrating that heterotetramers of CcIav‐CcNan, in conjunction with CcPKCα, precisely modulate CcAKHR expression at transcriptional, translational, and post‐translational levels (Figure 5A‐K). Acting as key downstream neuroendocrine effectors, CcAKH1 and CcAKHR orchestrated energy mobilization and absorption crucial for ovarian maturation in winter‐form females, thereby facilitating the phenotypic transition to summer‐form (Figure 6). Elevated environmental temperatures activate TRPV channels, triggering calcium influx that subsequently activates CcPKCα. Acting as a key regulatory factor, CcPKCα may modulate both: i) CcAKH1 synthesis/release in the head; ii) CcAKHR activity via phosphorylation in the fat body (as empirically demonstrated in this study). This integrated signaling cascade mediates robust temperature‐dependent activation of the AKH pathway, ultimately driving phenotypic transition. This study represents the first comprehensive evidence for AKH signaling in seasonal polymorphism, particularly in orchestrating the transition of pear psyllids from winter‐ to summer‐form. Analogous reports include dopamine signaling influencing the behavioral phase changes in migratory locust,^[^ ^42^ ^]^ insulin and its receptors determining alternative wing morphs in planthoppers,^[^ ^43^ ^]^ and neuropeptide Bursicon and its receptor mediating the transition from summer‐ to winter‐form in *C. chinensis*.^[^ ^10^ ^]^

**Figure 7:**
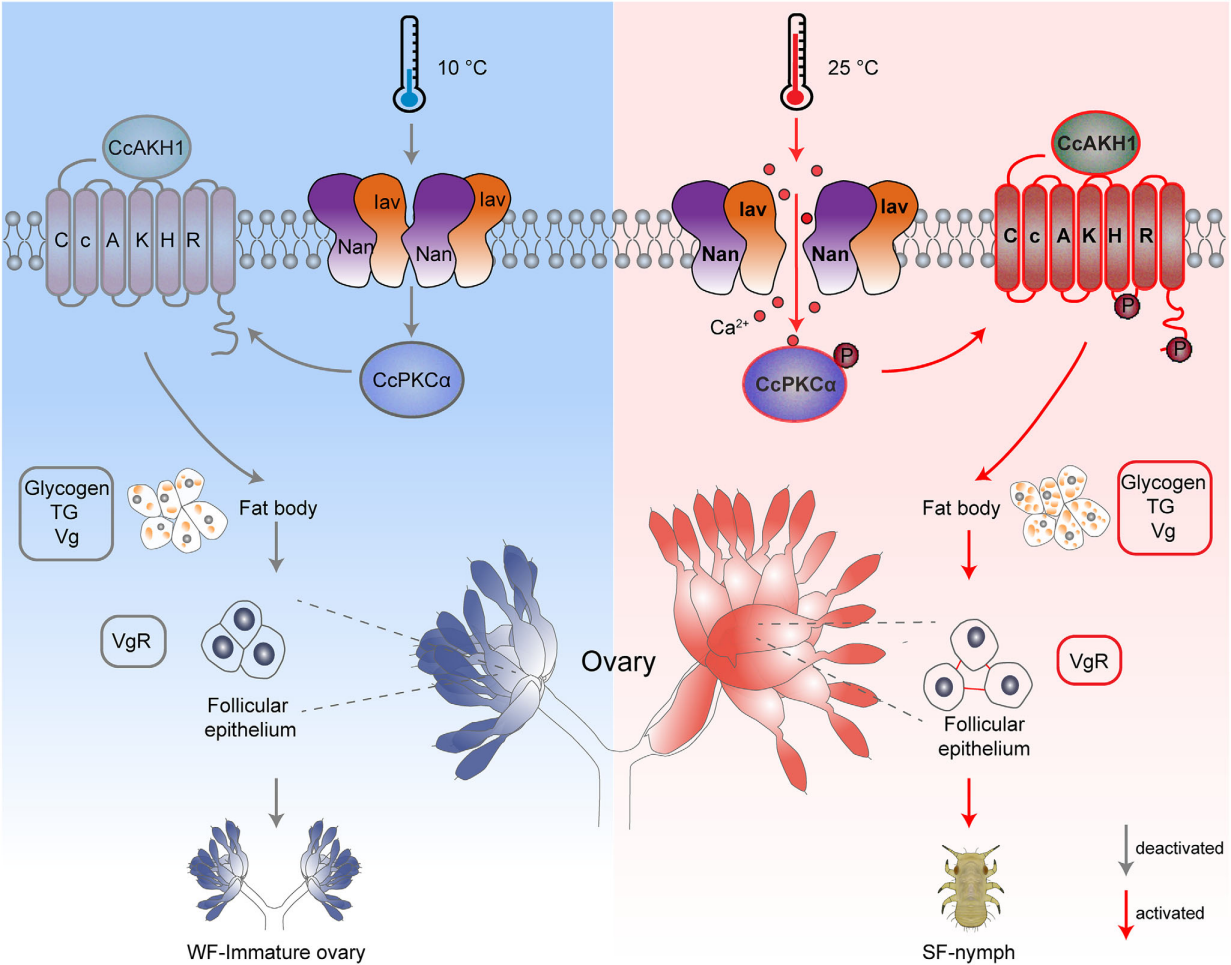
Molecular mechanism of CcIav/CcNan‐CcPKCα‐AKH/CcAKHR signaling axis governing temperature‐dependent transition from winter‐ to summer‐form in *C. chinensis*.

Given the significant evolutionary divergence in TRPV subfamily members between insects and mammals, three phylogenetically distinct orchard pests were selected to investigate the conservation of thermal response functions of Inactive and Nanchung. Our qRT‐PCR results demonstrated temperature‐dependent upregulation of *Inactive* and *Nanchung* transcripts at 25 °C across all species, while complementary protein and cellular data suggested a conserved mechanism involving heterotetramer formation for temperature/capsaicin sensing (Figure S16, Supporting Information). Collectively, we propose a novel signaling cascade (CcIav/CcNan‐CcPKCα‐CcAKH1/CcAKHR) regulating the transition from winter‐ to summer‐form in *C. chinensis* through coordinated regulation of energy mobilization and ovary development, as summarized in **Figure** **7**. Under low temperature conditions (10 °C), the thermal sensors *CcIav* and *CcNan* remain inactive, failing to initiate a calcium‐dependent signaling through CcPKCα and CcAKH/CcAKHR. This resulted in arrested ovarian development and blocked energy allocation in winter‐form females, preventing summer‐form nymph production. Contrastingly, exposure to 25 °C activates *CcIav*/*CcNan* heterotetramers assembly, triggering calcium influx that stimulates phosphorylation cascades in CcPKCα and CcAKHR. These molecular events enhanced energy substances mobilization and vitellogenin transport through modified follicular epithelium patency, ultimately driving rapid ovarian maturation and summer‐form nymph production, enhancing adaptability to the environment and fostering population prosperity. Future research endeavors will focus on two emerging aspects: i) epigenetic regulation (DNA methylation and histone acetylation) of thermal receptor‐mediated transition from winter‐ to summer‐form in *C. chinensis*, and ii) potential temperature‐gut microbiome‐ neuroendocrine interaction coordinating seasonal polyphenism of *C. chinensis*. These directions promise to deepen our understanding of environmental adaptation mechanisms in agricultural pests.

## 4 Experimental Section

### Insect Colonies and Host Plants

A founding colony of *C. chinensis* population was established from individuals collected in a pear orchard (Daxing District, Beijing; 39°43′N, 116°21′E) in September 2018. The insects were maintained through successive generations on grafted Korla fragrant pears seedlings (2‐3‐year‐old) under controlled environmental conditions: 25 ± 1 °C, 65 ± 5% relative humidity (RH), and 12 L: 12D photoperiod. Three additional arthropod systems were established for comparative analyses: i) *D. citri* population was collected from a citrus orchard in Zhanjiang, Guangdong (21°15′N, 110°24′E) in July 2021 and raised on healthy *Citrus limon* (L.) Osbeck plants at 26 ± 1 °C (65 ± 5% RH, 14L: 10D photoperiod). ii) *B. dorsalis* population was assembled in Fangshan, Beijing in July 2022, where both adults and larvae were nourished with artificial diets^[^ ^44^ ^]^ at 27 ± 1 °C (RH: 70 ± 5%, light cycle: 14L: 10D). iii) *H. axyridis* was gathered from a pear orchard in Fangshan, Beijing, in September 2021 and cultured on *Acyrthosiphon pisum*‐infected fava bean Vicia faba at 26 ± 1 °C (RH of 65 ± 5%, and a photoperiod of 14L: 10D).

### Temperature Induced the Seasonal Transition in *C. chinensis*

Building upon established protocols for cold‐induced winter‐form transition,^[^ ^2^, ^3^ ^]^ a reciprocal thermal regime was developed to study winter‐to‐summer induction: i) Winter‐form generation: summer‐form nymphs were acclimated at 10 ± 1 °C (65 ± 5% RH, 12L: 12D photoperiod) until adult emergence; ii) Thermal challenge: newly emerged winter‐form adults were transferred to climate chambers set at either 10 °C (diapause maintenance) or 25 °C (transition induction) while maintained on pear seedlings. Ovarian developmental trajectories were quantified through: Temporal dissection series: i) Ovaries were dissected from 30 females per time point (4, 7, 10, 15, and 20 days post‐temperature shift, with 3 biological replicates per time point); ii) Morphometric classification: Four‐stage developmental system adapted from Zhang et al. (2025) using stereo microscopy (Leica M205 FA)^[^ ^45^ ^]^; iii) Multi‐parametric analysis: bioenergetic markers (triglyceride and glycogen), structural dynamics (lipid droplet density and follicular patency index), molecular signatures (qRT‐PCR profiling of vitellogenin and its receptor mRNA expression).

### Quantification of Bioenergetic Markers and Ovarian Intercellular Channels

Ovaries' triglyceride (TG) content was quantified using the GPO‐PAP enzymatic system (Ca# A110‐1‐1, Jiancheng Biotechnology, Nanjing, China) following manufacturer specifications. Glycogen levels were measured via a glycogen assay kit (Ca# A043‐1‐1, Jiancheng Biotechnology). Lipid droplets distribution was visualized using Nile Red Fluorescent Staining (Ca# G1264, Solarbio, Beijing, China). TG content and glycogen levels were measured in 9 biological replicate samples with each from 30 pooled ovaries. Actin cytoskeleton architecture and intercellular channels were demarcated with Actin‐Tracker Green‐488 (1: 100 dilution in PBST/5% BSA, Ca# C2201S, Beyotime). The follicular patency index was calculated as the ratio of the intercellular space area surrounding a representative follicle cell to the average cross‐sectional area of follicle cells within the same image using ImageJ software.

### Gene Cloning and Structural Modeling

Full‐length sequences of *CcIav*, *CcNan*, *CcPKC*, *CcAKH*, and *CcAKHR* were retrieved from transcriptome and NCBI data, validated by PCR and RACE (Table S1, Supporting Information). Structural analyses included: i) Quaternary modeling: CcIav/CcNan heterotetramer predicted using AlphaFold3 (https://alphafoldserver.com/)^[^ ^46^ ^]^; ii) Molecular docking (AutoDock 4.2.6): global search (semi‐flexible parameters), Local optimization (flexible docking with Lamarckian GA), Top‐ranked poses (ΔG ≤ ‐7.5 kcal/mol, RMSD<2.0 Å threshold); iii) Additional predictions: 1) CcPKCα monomer topology and CcAKH1/CcAKHR interfaces (AlphaFold3); 2) conserved domains (SMART database); 3) Transmembrane helix prediction (TMHMM v2.0). Homologous protein sequences were obtained via NCBI Blast and aligned using DNAMAN 9.0 software. Phylogenetic reconstruction (MEGA7, maximum likelihood method, 1000 bootstraps) incorporated 36 insect TRPV homologs. Putative PKC phosphorylation sites on CcAKHR were predicted using NetPhos3.1. Site‐directed mutagenesis at high‐probability residues was performed using Mut Express II Fast Mutagenesis Kit (Cat# C214, Vazyme) (Table S1, Supporting Information). Mutant *CcAKHR* was utilized for calcium ion detection and in vitro kinase reactions.

### qRT‐PCR Analysis and Functional Interference Assays

To delineate temperature‐responsive transcriptional dynamics, winter‐form females were collected at each time point (*n* = 3 biological replicates; each from 30 pooled females). Tissue‐specific profiling was conducted through microdissection of seven anatomical structures from females (n = 3 biological replicates; each from 50 females acclimated 7 days). RNA was extracted using the MiniBEST Universal RNA Extraction Kit (Ca# 9767, TaKaRa, Japan), followed by cDNA synthesis via PrimeScript RT reagent Kit (Ca# RR047A, TaKaRa) and analyzed by qRT‐PCR (TB Green Premix Ex Taq, TaKaRa) with 3 technical replicates. The reference genes *Ccβ*‐actin and *CcEF1* were used for normalization (primers listed in Table S1, Supporting Information). Data analysis was performed using the 2^−ΔΔCT^ method.^[^ ^47^ ^]^

Target‐specific dsRNA (1000 ng/µL) was synthesized using MEGAscript T7 kit (Ca# AM1334, Thermo Fisher, USA) with T7‐flanked primers (Table S1, Supporting Information). For functional studies: i) Preconditioning: newly emerged winter‐form adults were maintained at 25 °C for 5 days to initiate phenotypic transition; ii) Compound exposure: 3‐day continuous dsRNA feeding via the stem‐leaf apparatus.^[^ ^1^ ^]^ Co‐treatment regimens including TRPV agonist capsaicin (Ca# HY‐10448, MedChemExpress, Shanghai, China, 1, 10, 100 µm), TRPV antagonist SB‐366791 (Ca# HY‐12245, MedChemExpress, 1, 10, 100 µm), calcium modulators ionomycin (Ca# HY‐13434, MedChemExpress, 1 µm) and EGTA (Ca# ST068, Beyotime, 50 nm); iii) Phenotypic assessment: At 72 h post‐treatment, molecular efficacy was quantified (target gene suppression via qRT‐PCR), morphological outcomes (ovarian developmental stages, lipid droplet distribution, follicular patency index, and reproductive fitness. Reproductive fitness, defined as the number of summer‐form offspring produced, was assessed by recording the average number of offspring hatched per 10 females (n = 6 biological replicates; each from distinct female groups). For the Egg dsRNA application, a 1 µL droplet (1000 ng µL^−1^) was applied to individual eggs (≥30 eggs per replicate). Post‐treatment samples were collected for: i) hatch rate observation, and ii) RNA extraction/gene expression analysis, with each treatment maintained across six biological replicates.

### Protein Interaction and Phosphorylation Analysis—*Molecular Cloning & Transfection*

Target ORFs were subcloned into pCDNA3.1‐mCherry‐P2A vectors, and 1 µg recombinant plasmids were transfected into HEK293T cells in a 24‐well plate using 3 µL of StarFect Lip2000 transfection reagent (Cat# C105, GenStar, Beijing, China). In co‐transfection experiments, the plasmid ratios were set as follows: for dual transfection of CcIav and CcNan, 1:2, 1:1, and 2:1; for quadruple transfection of CcIav, CcNan, CcPKCα, and CcAKHR, 1:1:1:1.

### Protein Interaction and Phosphorylation Analysis—*Protein Extraction & Quantification*

After 48‐h, cells were lysed in ice‐cold RIPA buffer (Cat# P0013, Beyotime) supplemented with 1 mm PMSF (Cat# ST506, Beyotime) and 1 × protease inhibitor cocktail (Cat# P1005, Beyotime). Lysates were sonicated (3 × 10 sec pulses), centrifugation (12000 × g, 15 min, 4 °C), and quantified via BCA assay (Cat# P0012S, Beyotime).

### Protein Interaction and Phosphorylation Analysis—*Electrophoretic Separation & Immunoblotting*

Proteins were denatured (95 °C, 10 min) in 6 × SDS‐PAGE Loading Buffer (Cat# DL101, Transgen, China) and resolved on 8% (CcIav, CcNan, and CcPKCα) or 10% (CcAKHR and β‐actin) gels. PVDF membranes (Merck Millipore, USA) were blocked with 5% non‐fat milk for 1 h. Membranes were probed with: Primary antibodies (1: 3000) including Mouse anti Flag, ‐HA, ‐V5, ‐Myc (ABclonal, China); HRP‐Conjugated secondary antibodies (1: 5000): Goat Anti‐Mouse IgG (H+L) (Transgen); Internal control: β‐Actin Mouse mAb (Transgen). Signals were detected with Clarity Western ECL Substrate (Bio‐Rad, USA) on Azure C600.

### Protein Interaction and Phosphorylation Analysis—*Native‐PAGE Analysis*

Non‐denatured proteins were separated^[^ ^48^ ^]^ using: 5 × Native‐PAGE protein loading buffer (Ca# P1042, Solarbio), Tris‐Glycine buffer (pH 8.3) without SDS/β‐ME. Post‐transfer processing followed standard immunoblot protocols.

### Protein Interaction and Phosphorylation Analysis—*Subcellular Fractionation*

Membrane/cytoplasmic fractions were isolated from HEK293T cells pooled from two wells of a 24‐well plate per replicate (n = 3) using a specialized kit (Cat# P0033, Beyotime): i) Hypotonic lysis (buffer A: 0.32 m sucrose, 5 mm Tris‐HCl, 120 mm KCl, 1 mm EDTA, PMSF); ii) Differential centrifugation: 700 × g (10 min, 4 °C) to pellet nuclei and cell debris, 14 000 × g (30 min, 4 °C) to separate membrane fragments (pellet) from cytoplasmic proteins (supernatant); iii) Membrane solubilization: Pellet resuspended in Buffer B (20 mm HEPES, 10% glycerol, 2% Triton X‐100, 1 mm EDTA, 1 mm EGTA, PMSF) with sonication (3 × 10 sec pulses).

### Protein Interaction and Phosphorylation Analysis—*Co‐Immunoprecipitation (Co‐IP)*

i) Treatment: Cells were exposed to 100 µm capsaicin (in 0.1% DMSO) or EGTA gradient (0.1–10 mm in PBS) for 10 min (37 °C). ii) Lysis: RIPA buffer + phosphatase inhibitors (Ca# P1045, Beyotime). iii) Immunoprecipitation: 500 µL lysate + Protein A/G magnetic beads (Ca# P2108, Beyotime) pre‐bound with 1 µg Anti‐Tag Rabbit mAbs (Ca# AE092, AE105, AE089, AE070, ABclonal). IgG (Ca# PP64B, Merck Millipore) as a negative control. iv) Elution: Boiling in 2 × SDS‐PAGE loading buffer at 70 °C for 10 min. v) Detection: Immunoblotting with mouse mAb (1: 3000). Empty vectors were used, including a control transfected with only one bait/target and another empty vector control, to demonstrate specificity beyond IgG.

### Protein Interaction and Phosphorylation Analysis—*Phosphorylation Profiling*

After Co‐IP, the protein phosphorylation levels were assessed using a 5% BSA solution for blocking and antibody dilution, with primary pan Phospho‐Serine/Threonine mAb at a 1:1000 dilution (Cat# AP1067, ABclonal), and Goat Anti‐Mouse IgG (H+L) secondary antibody in a 5% BSA solution.

### Protein Interaction and Phosphorylation Analysis—*In Vivo Protein Validation*

Procedures mirrored in vitro experiments with slight modifications: i) Primary antibodies: PKCα Rabbit pAb (1:2000, Ca# 21991‐1‐AP, Proteintech, Wuhan, China) and AKHR Rabbit pAb (1:2000)^[^ ^29^ ^]^; ii) Secondary antibody: HRP‐conjugated Mouse anti‐Rabbit IgG Light Chain (1:5000, Ca# AS061, ABclonal). Protein extracts were prepared from pools of 30 females per treatment, with three independent biological replicates.

### In Vitro Kinase Assay and Ca^2+^ Imaging

Recombinant CcPKCα‐V5‐His and CcAKHR‐Myc‐His proteins were affinity‐purified using a His‐tagged protein purification kit (Cat# P2226, Beyotime). The kinase reaction system (20 mm HEPES PH 7.4, 1.67 mm CaCl_2_, 1 mm DTT, 10 mm MgCl_2_, 200 µm ATP, 1 µm PMA) contained 50 ng CcPKCα and 25 ng CcAKHR. Reactions were terminated after 30 min incubation at 30 °C by adding SDS‐PAGE loading buffer for subsequent western blot analysis.^[^ ^49^ ^]^

To explore ligand effects on receptor activation, Ca^2+^ imaging and fluorescence‐based Ca^2+^ concentration measurement techniques were employed as previously described.^[^ ^1^ ^]^ Capsaicin‐binding site mutants were generated in pCDNA3.1‐mCherry‐P2A‐CcIav through site‐specific mutagenesis based on molecular docking predictions. HEK293T cells in 96‐well plates (0.25 µg plasmid per well) or confocal dishes (1 µg plasmid per dish) were transfected using StarFect Lip2000 Transfection Reagent (at a 1:3 ratio). After transfection, cells were incubated with the Fluo‐4 AM probe (Cat# S1061, Beyotime, China) for 30 min. Subsequent stimulation with ligands was followed by Ca^2+^ imaging and concentration measurement utilizing a Leica SP8 confocal microscope and MD i3x microplate reader. Capsaicin gradient: 10 to 5 mm (in 0.1% DMSO) for Ca^2+^ concentration assessment. A concentration of 100 µm capsaicin was specifically employed for Ca^2+^ imaging in the context of the CcIav mutant. CcAKH1 mature peptide (Sangon Biotech): 0.1 nm to 10 µm (in water) for Ca^2+^ concentration evaluation. The measurement involving CcAKHR mutants used a mature peptide of CcAKH1 at 100 nm. Changes in fluorescence (ΔF/F_0_) were employed to signify alterations in Ca^2+^ levels, with F_0_ denoting baseline fluorescence and ΔF indicating the stimulation‐induced variance. Each treatment in the Ca^2+^ concentration measurement was subjected to 6 or 9 biological replicates.

### Fluorescence In Situ Hybridization (FISH) and Subcellular Localization

Gene‐specific fluorescent probes (CcIav‐Cy3: 5′‐ TGGGTGCACATGGAATTAGG‐ 3′; CcNan‐FAM: 5′‐ TGAGGAACATCGCCATGATG‐ 3′) were used following this protocol: i) Fixation in Carnoy's solution for 24 h; ii) Decolorization in 6% hydrogen peroxide in ethanol for 48 h; iii) Pre‐hybridization in probe‐free buffer (20 mm Tris‐HCl pH 8.0, 0.9 m NaCl, 0.01% sodium dodecyl sulfate, 30% formamide) and probes hybridization (2 µm) for 24 h; 4) Post‐hybridization washes, DAPI staining, and confocal microscopy (Leica SP8).

For subcellular co‐localization, HEK293T cells transfected with CcIav‐Flag and CcNan‐Myc were cloned into the pCMV‐SV40‐Neo vector, then i) fixed with 4% PFA and permeabilized with ice‐cold methanol, ii) blocked with 5% BSA, iii) incubated with primary antibodies (Rabbit anti‐Flag mAb and Mouse anti‐HA mAb, 1:500), iv) stained with secondary antibodies (Cy3‐conjugated Goat anti‐Rabbit IgG H+L, AF488‐conjugated Goat anti‐Mouse IgG H+L, 1:200, ABclonal), v) visualized by confocal microscopy.^[^ ^50^, ^51^ ^]^

For subcellular CcAKHR localization, HEK293T cells transfected with pEGFP‐3×Linker CcAKHR were, i) fixed with 4% PFA after 48 h, ii) stained with Dil (Ca # C1036, Beyotime) and DAPI,^[^ ^52^ ^]^ iii) imaged by confocal microscope.

### Statistical Analysis

Results are presented as mean ± standard error (SE) from different replicates (n = 6 for transition percent; n = 9 for qRT‐PCR data, and cell assays; n = 12 for patency index). Data analysis performed in GraphPad Prism 8.0 software included: i) Two‐group comparisons: unpaired Student's *t*‐test (^*^ *p* < 0.05, ^**^ *p* < 0.01, ^***^ *p* < 0.001, ns: not significant); ii) multi‐group analysis: one‐way ANOVA with Tukey's HSD test (differing letters indicate *p* < 0.05).

## Author Contributions

S.Z. designed research; S.Z. and J.L. performed research; Z.Z., Y.W., Y.Y., J.W., and X.R. contributed new analytic tools; S.Z. and J.L. analyzed data; S.Z. and J.L. wrote the paper; S.Z. provided the funds.

## Conflict of Interest

The authors declare no conflict of interest.

## Supporting information



Supporting Information

## Data Availability

The data that support the findings of this study are available in the supplementary material of this article.
