# Supplementary material for: Thermosensory TRPV Heterotetramers Drive Seasonal Polyphenism: Molecular Basis of CcIav/CcNan‐PKCα‐AKH/AKHR Signaling in Pear Psyllid Morph Transition
Source: Adv Sci (Weinh). 2025 Sep 15;12(45):e10102. doi: 10.1002/advs.202510102 (PMC12677633; doi:10.1002/advs.202510102)
Supplement: Supplementary file 1 — Supporting Information [file ADVS-12-e10102-s001.docx]

**Supporting Information**

**Thermosensory TRPV Heterotetramers Drive Seasonal Polyphenism: Molecular Basis of CcIav/CcNan-PKCα-AKH/AKHR Signaling in Pear Psyllid Morph Transition**

*Jianying Li, Zhixian Zhang, Yilin Wang, Yue Yang, Jiarui Wang, Xueyi Ruan, and Songdou Zhang**

**
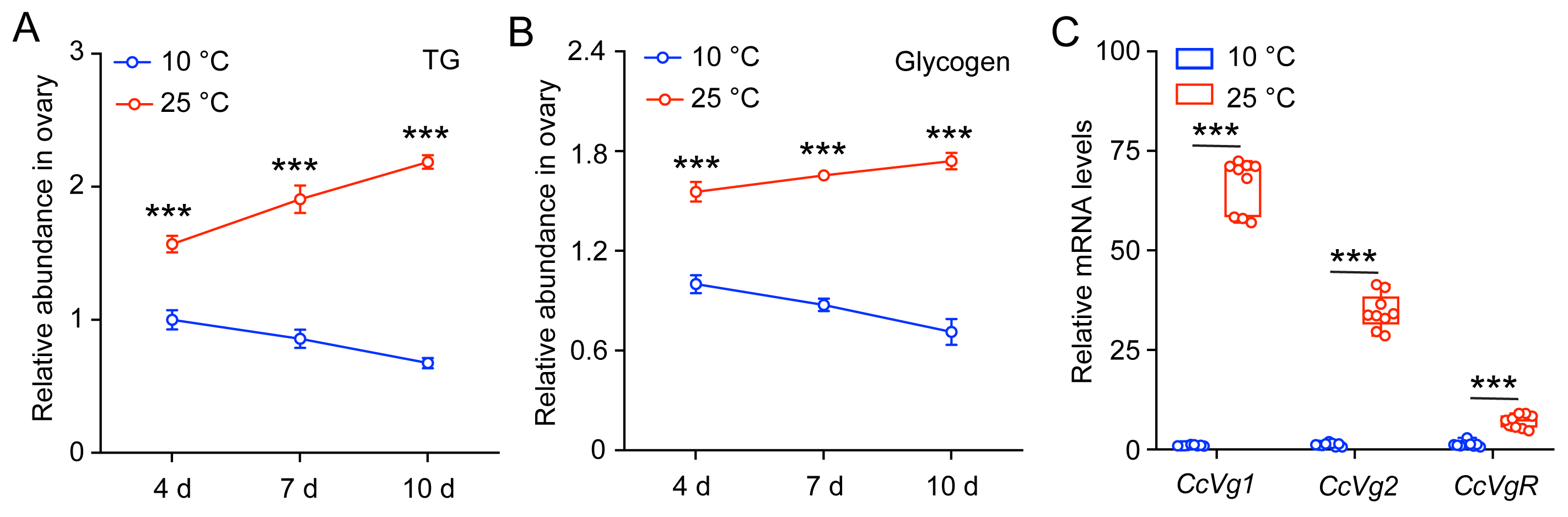
**

**Figure S1. Temperature-dependent regulation of metabolic and vitellogenesis markers in newly emerged winter-form females.**

Triglyceride levels, glycogen content, and mRNA expression of *CcVg1*, *CcVg2*, and *CcVgR* in newly emerged winter-form females under 10 °C vs. 25 °C at indicated timepoints. Data: mean ± SEM (n=9; ≥30 insects/replicate). ****p*<0.001 (Student’s *t*-test).

**
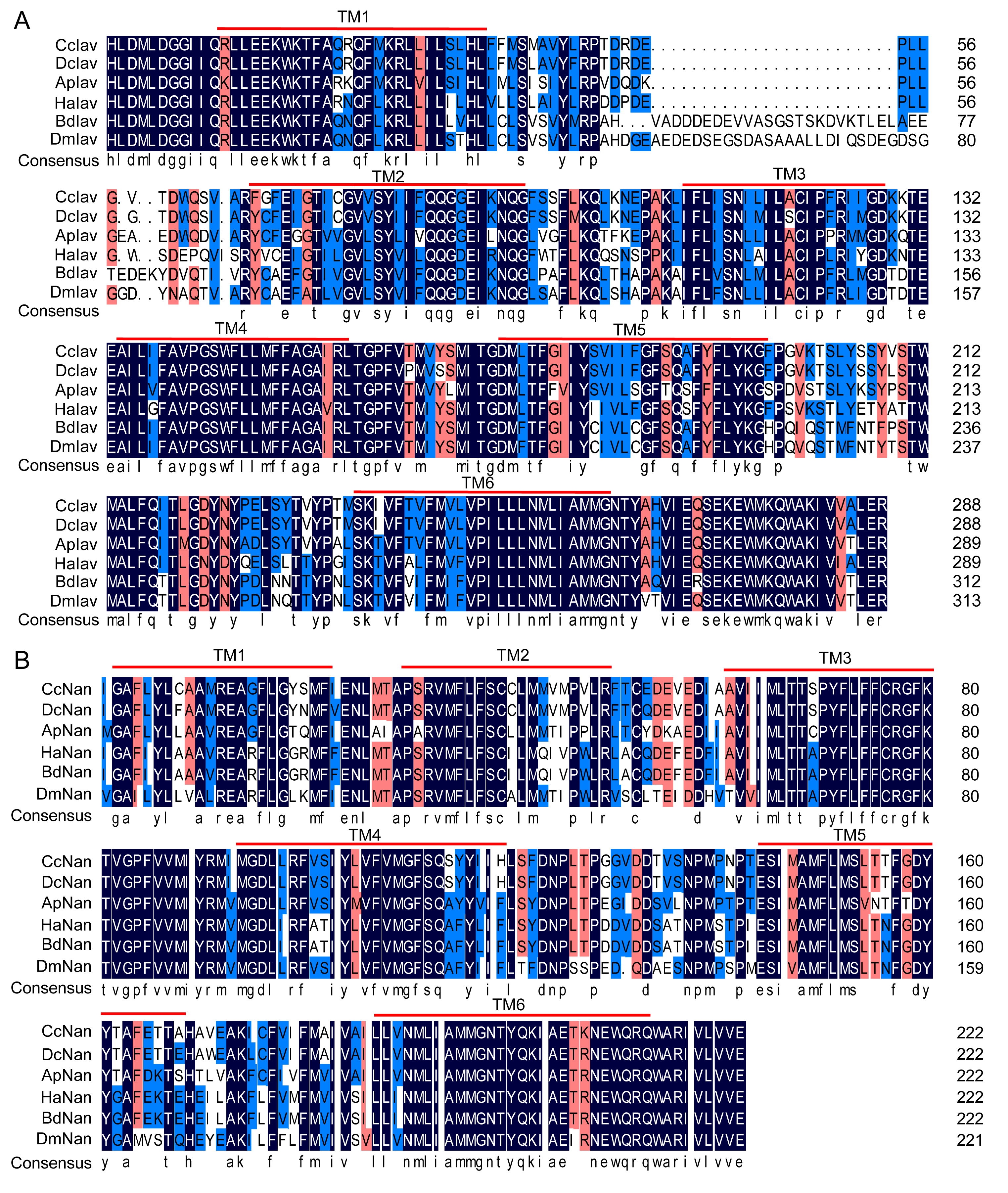
**

**Figure S2. Conserved transmembrane architecture of CcIav/CcNan across six insect species.**

Multiple sequence alignment of transmembrane domains (TM1-TM6, red bars) for TRPV subunits. Top: *CcIav* (*C. chinensis*, PQ818806), *DcIav* (*Diaphorina citri*, PQ818822), *ApIav* (*Acyrthosiphon pisum*, XP_001950096.1), *HaIav* (*Harmonia axyridis*, PQ818826), *BdIav* (*Bactrocera dorsalis*, PQ818824), and *DmIav* (*Drosophila melanogaster*, NP_572353.1). Bottom: *CcNan* (*C. chinensis*, PQ818807), *DcNan* (*D. citri*, PQ818823), *ApNan* (*A. pisum*, XP_016658292.1), *HaNan* (*H. axyridis*, PQ818827), *BdNan* (*B. dorsalis*, PQ818825), and *DmNan* (*D. melanogaster*, NP_648696.2).

**
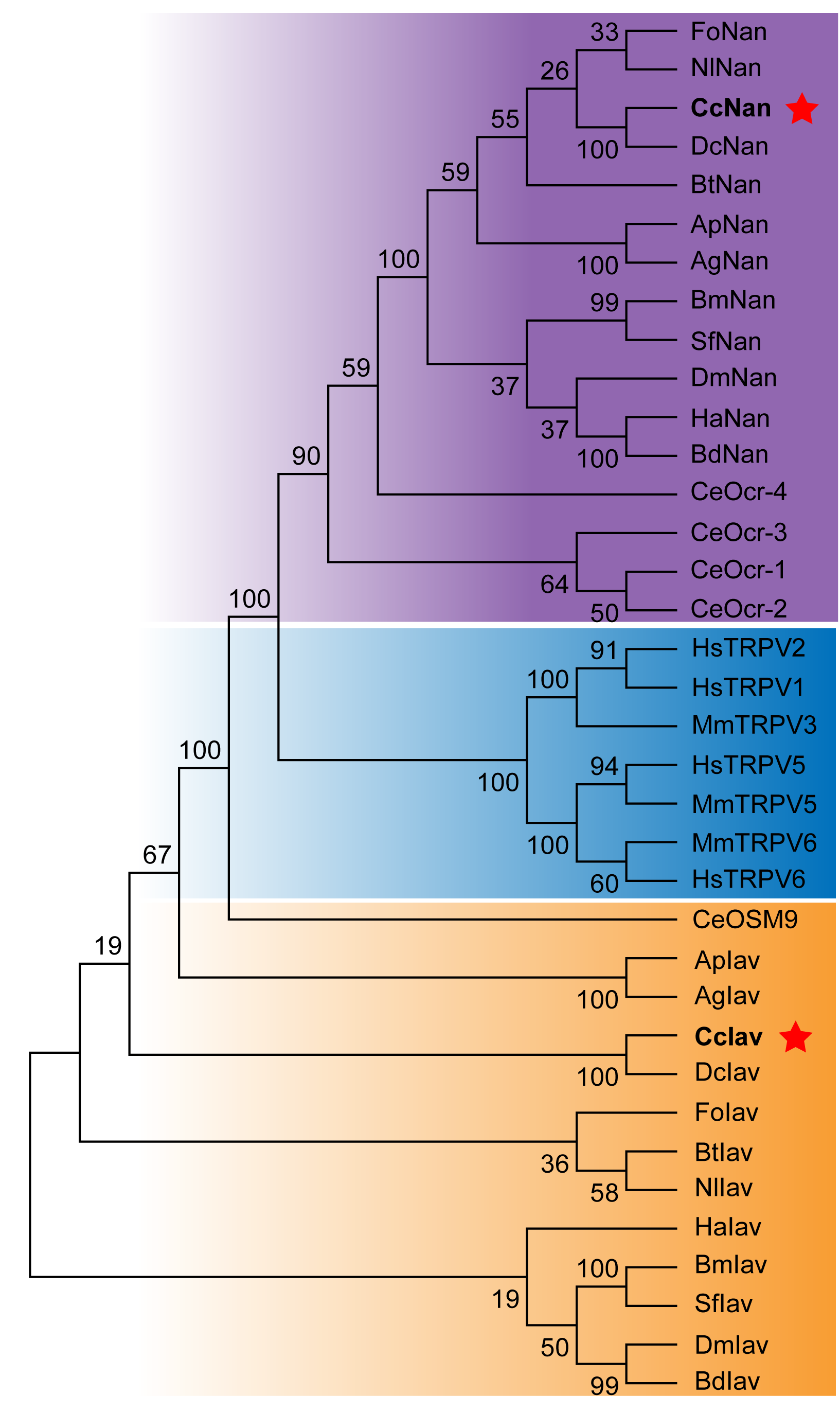
**

**Figure S3. Phylogenetic relationships of insect TRPV homologs.**

Maximum likehood tree of CcIav and CcNan (highlighted by red stars) with orthologs from representative species. Accession numbers provided in Table S2.


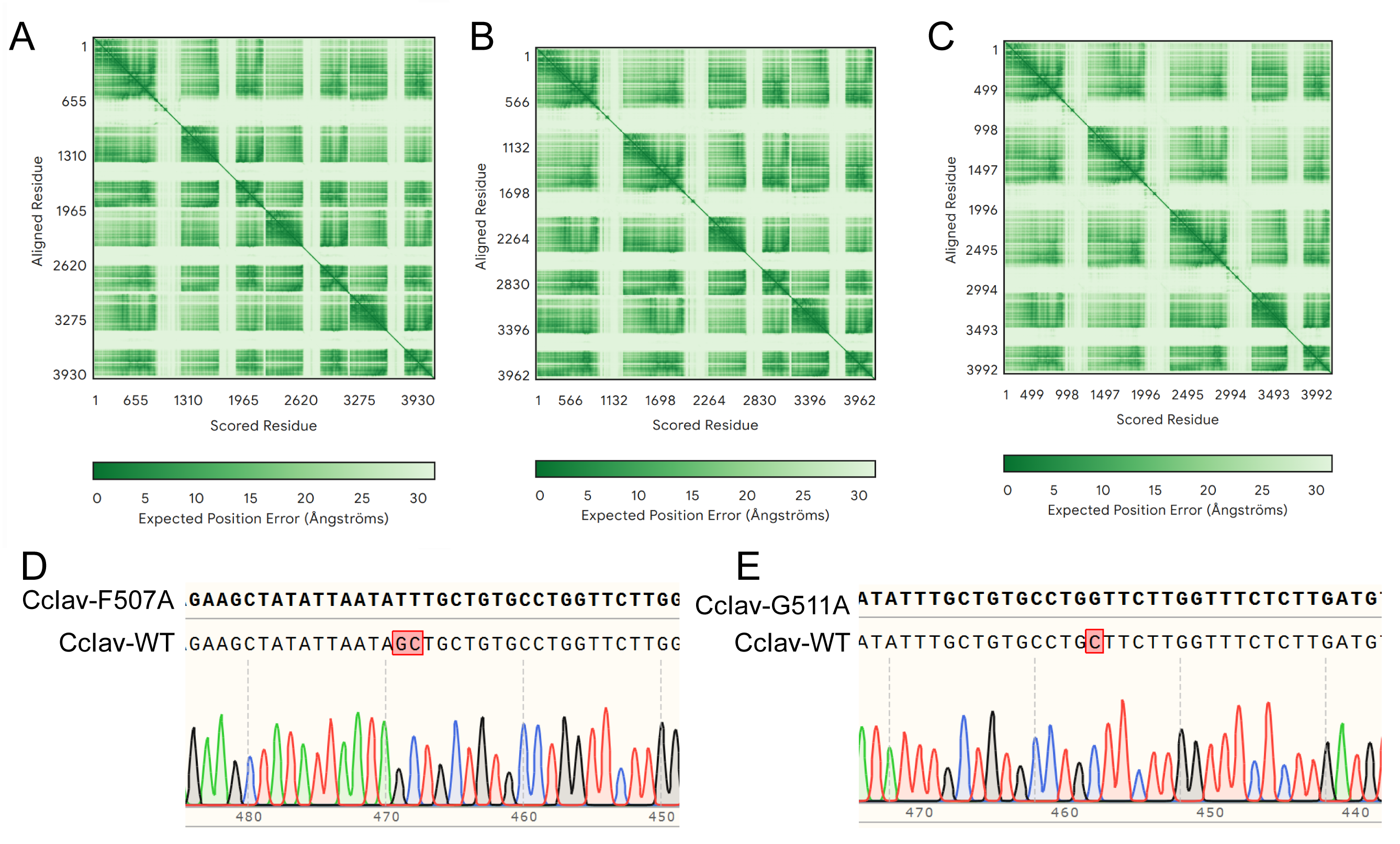


**Figure S4. Structural validation of CcIav/CcNan interaction models and mutagenesis.**

A-C: Alphafold3-predicted residue scores for the heterotetrameric structures of CcIav and CcNan at the molecular ratio of 3:1 (ipTM=0.63, pTM=0.64), 2:2 (ipTM=0.67, pTM=0.67), and 1:3 (ipTM=0.64, pTM=0.66). D-E: Sanger sequencing validation of CcIav site-directed mutation.


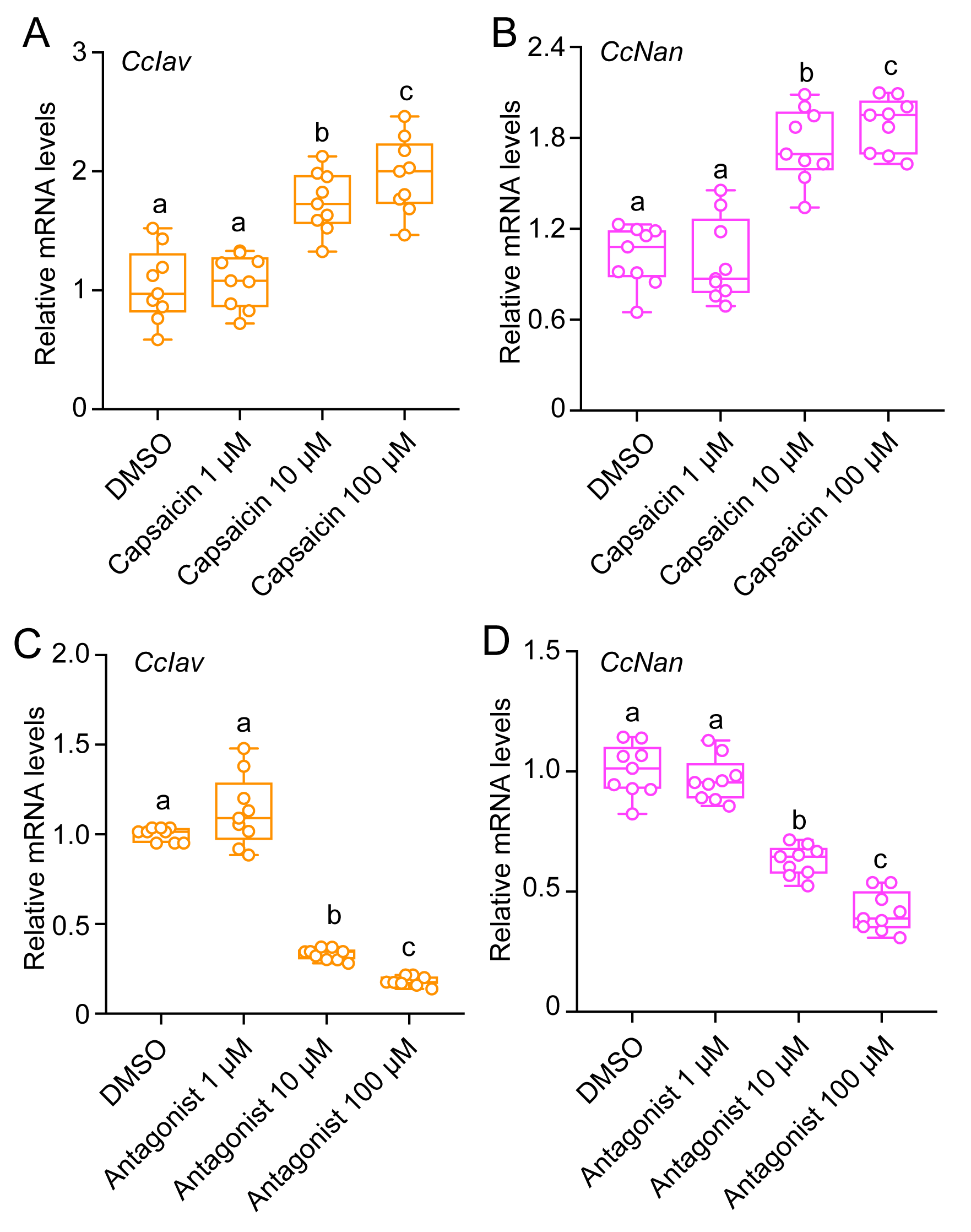


**Figure S5. Pharmacological modulation of *CcIav*/*CcNan* expression profiles.**

A-B: Dose-responsive *CcIav* and *CcNan* mRNA changes under capsaicin (vs. DMSO control). C-D: TRPV antagonist (SB-366791) suppression of subunit expression. Data: mean ± SEM (n= 3 biological × 3 technical replicates). Letters denote significance (ANOVA/Tukey’s HSD; *p* < 0.05).


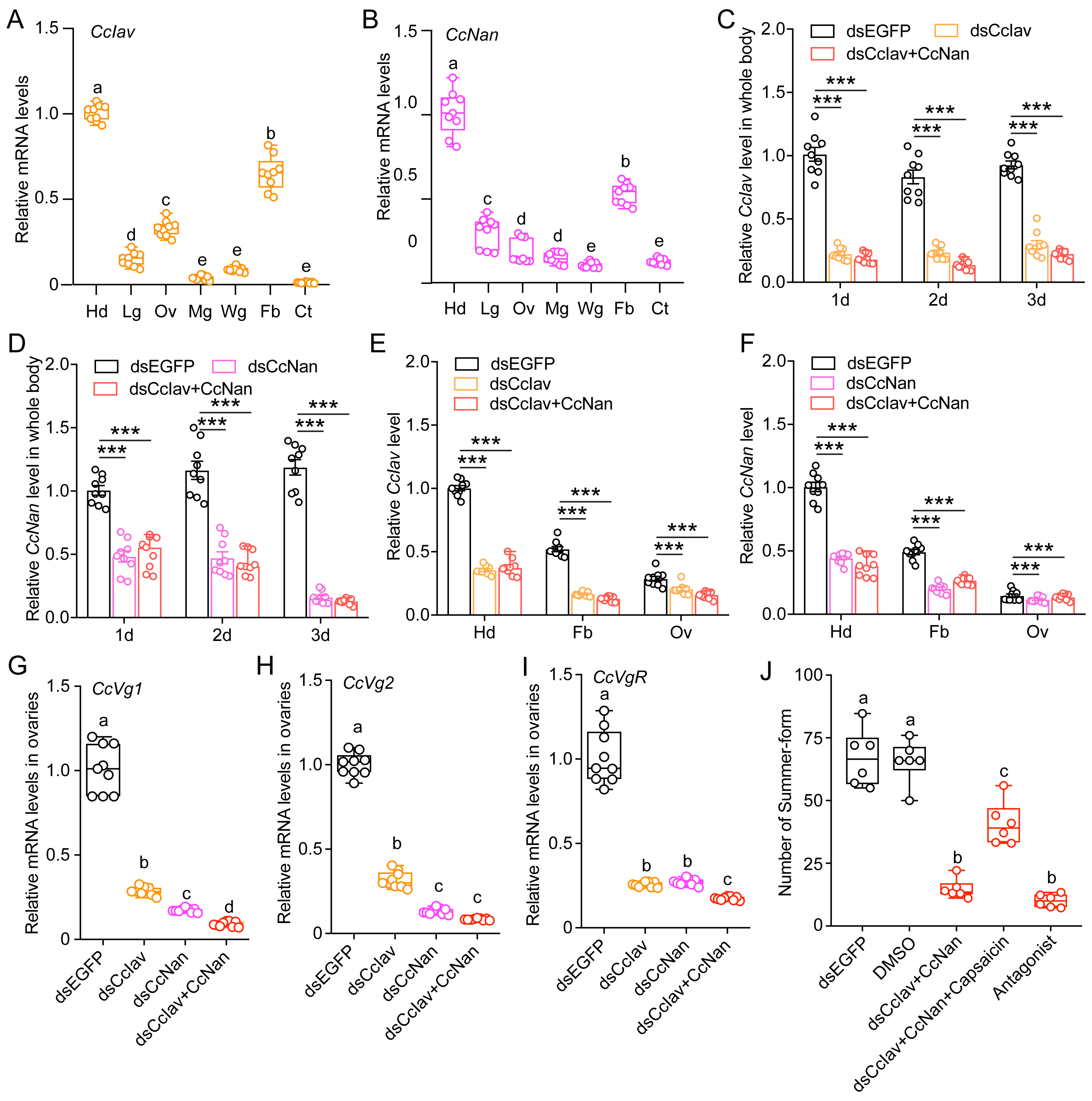


**Figure S6.** **Tissue-specific expression and functional analysis of CcIav/CcNan in seasonal transition regulation.**

A-B: qRT-PCR analysis of *CcIav* and *CcNan* across tissues. Hd: head, Lg: leg, Ov: ovary, Mg: midgut, Wg: wing, Fb: fat body, Ct: cuticle. C-F: Assessment of RNAi efficiency targeting *CcIav* and *CcNan* in winter-form females for temporal knockdown (1-3 days post-treatment) and tissue-specific suppression (3 days). G-I: Analysis of mRNA expression levels of *CcVg1*, *CcVg2*, and *CcVgR* following treatments with dsCcIav*,* dsCcNan, and combined knockdown at 3 days. J: The number of summer-form nymph transitioning from winter-form females following treatments with dsEGFP, DMSO, dsCcIav, dsCcNan, capsaicin (10 μM) a TRPV antagonist (10 μM). Data presentation of A-I: Mean ± SEM (n=3 biological × 3 technical replicates) and J: Mean ± SEM (n=6 biological replicates). Statistical analysis: Letters above the bars denote significant differences (ANOVA/Tukey’s HSD, *p* < 0.05), ****p*<0.001 (Student’s *t*-test).


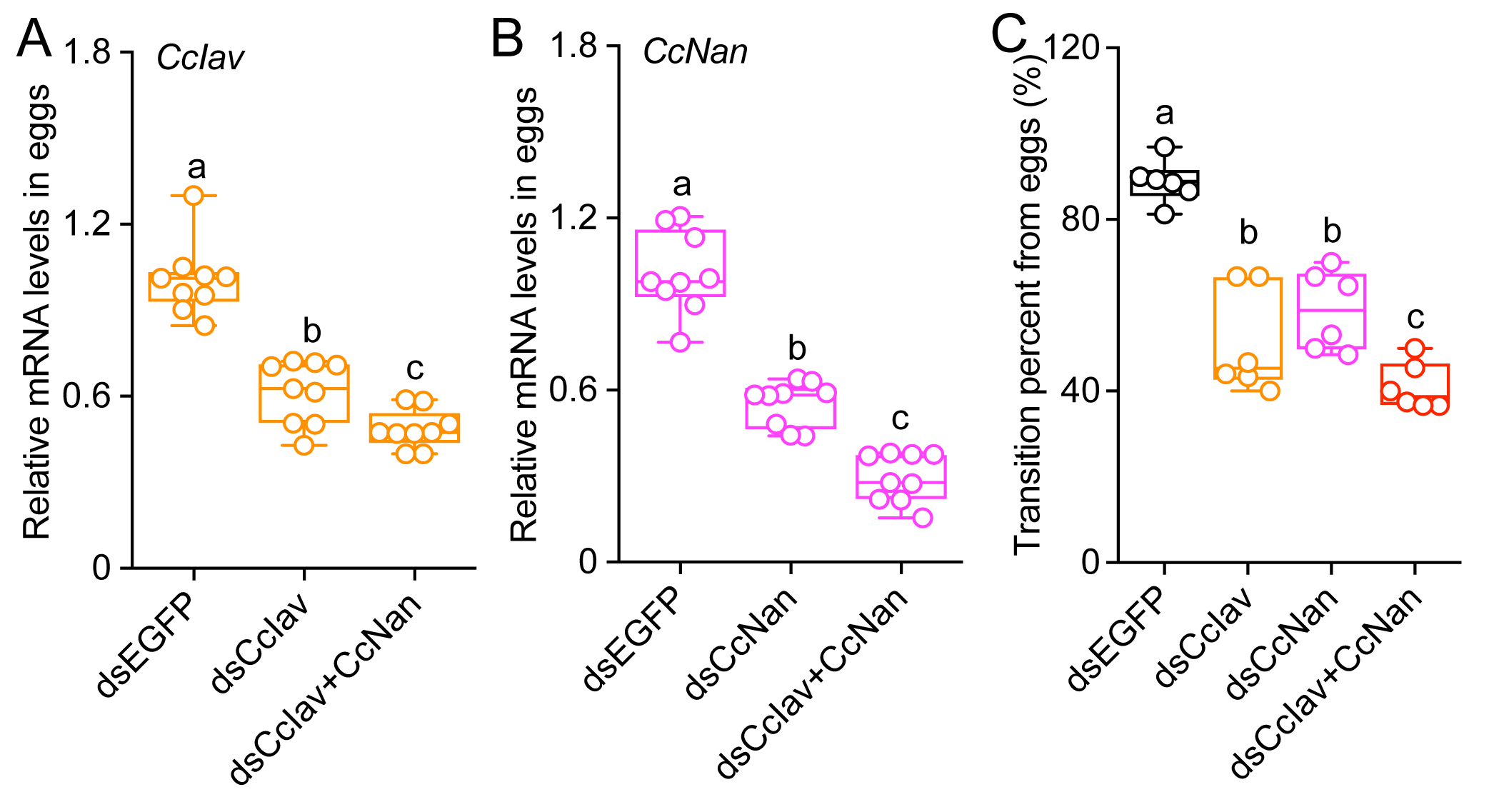


**Figure S7. RNAi efficiency and phenotypic impact on offspring transition.**

A-B: *CcIav* and *CcNan* transcript suppression in summer-form eggs from dsRNA-treated winter-form adults. C: Impaired egg-to-summer-form nymph transition rate post-RNAi. Data: mean ± SEM (A-B: n=3 biological × 3 technical replicates ; C: n=6). Letters denote significance (ANOVA/Tukey’s HSD; *p* < 0.05).


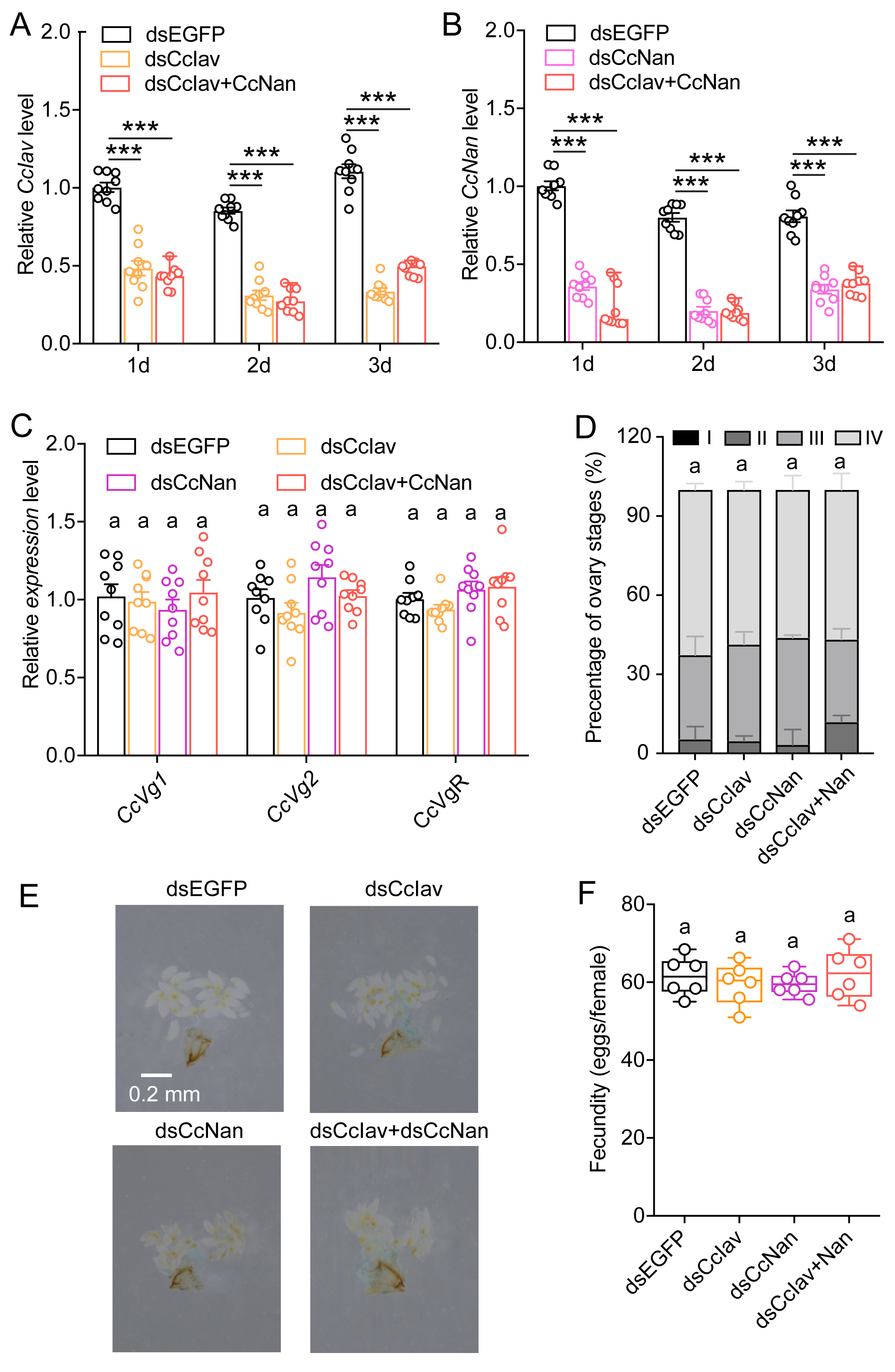


**Figure S8. Effect of *CcIav*/*CcNan* knockdown on the ovary development of summer-form females.**

A-B: Temporal analysis of RNAi efficiency targeting *CcIav* and *CcNan* in 5-day-old summer-form females at 1, 2, and 3 days post-dsRNA treatment. C: Downregulation effects of *CcIav*, *CcNan*, or combined knockdown on vitellogenesis-related gene expression (*CcVg1*, *CcVg2*, and *CcVgR*). D-F: Phenotypic consequences of ovarian development and fecundity after gene knockdown in 5-day-old summer-form females.

Data presentation of A-C: Mean ± SEM (n=3 biological × 3 technical replicates), D: Mean ± SEM (n=3 biological replicates) and F: Mean ± SEM (n=6 biological replicates). Statistical analysis: Letters above the bars denote significant differences (ANOVA/Tukey’s HSD, *p* < 0.05), ****p*<0.001 (Student’s *t*-test).

**
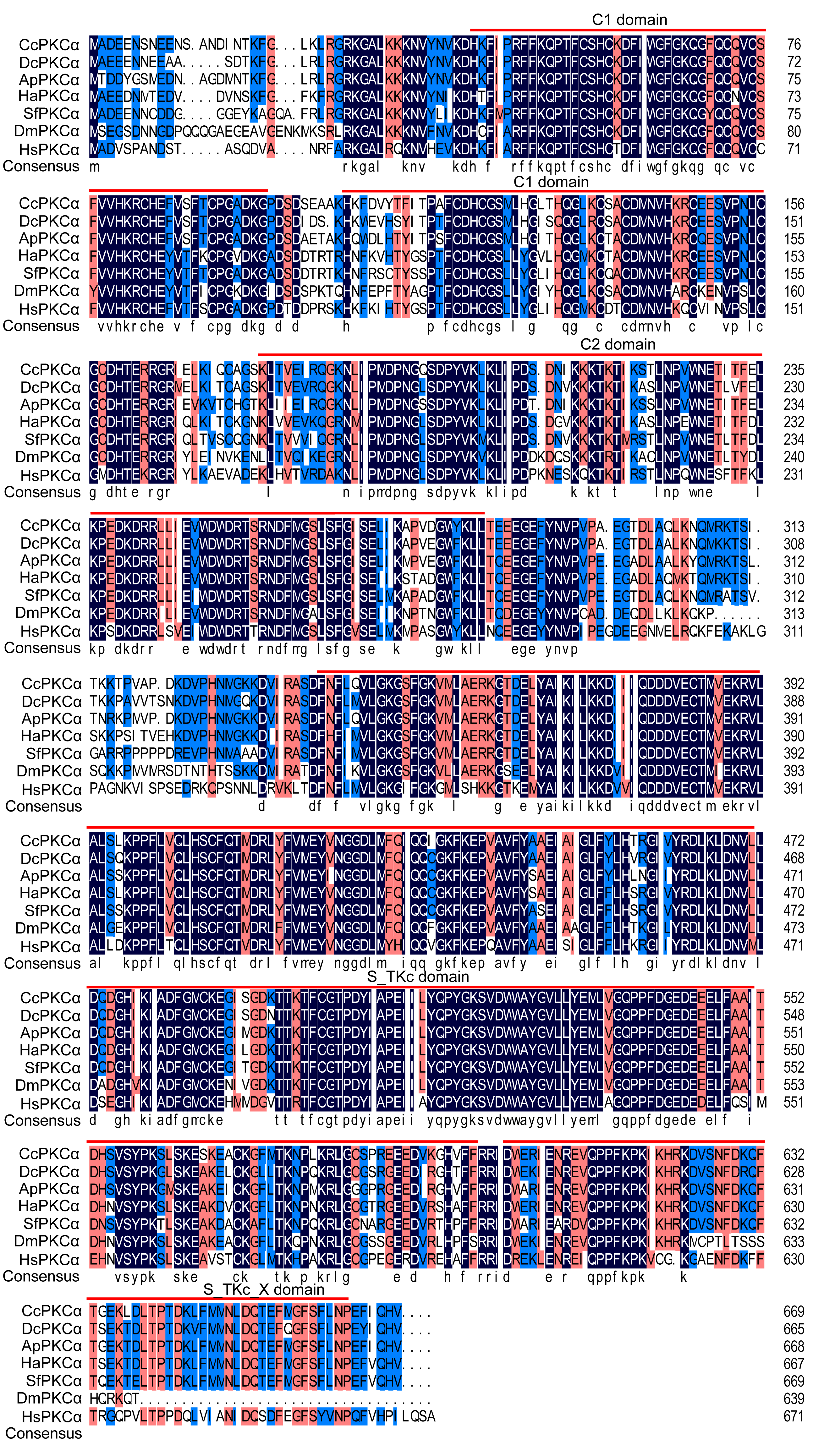
Figure S9.** **Conserved domain architecture of PKCα across seven species.**

Multiple sequence alignment highlighting functional domains (red bars): C1 (conserved region 1), C2 (conserved region 2, Calcium binding domain), S_TKc (Serine/Threonine catalytic domain), and S_TKc_X (kinase extension domain). *CcPKCα* (*C. chinensis*, PQ818811), *DcPKCα* (*D. citri*, XP_026685144.1), *ApPKCα* (*A. pisum*, XP_001945397.2), *HaPKCα* (*H. axyridis*, XP_045465229.1), *SfPKCα* (*S. frugiperda*, XP_035440495.1), *DmPKCα* (*D. melanogaster*, CAA28890.2), *HsPKCα* (*H. sapiens*, BAU98542.1).


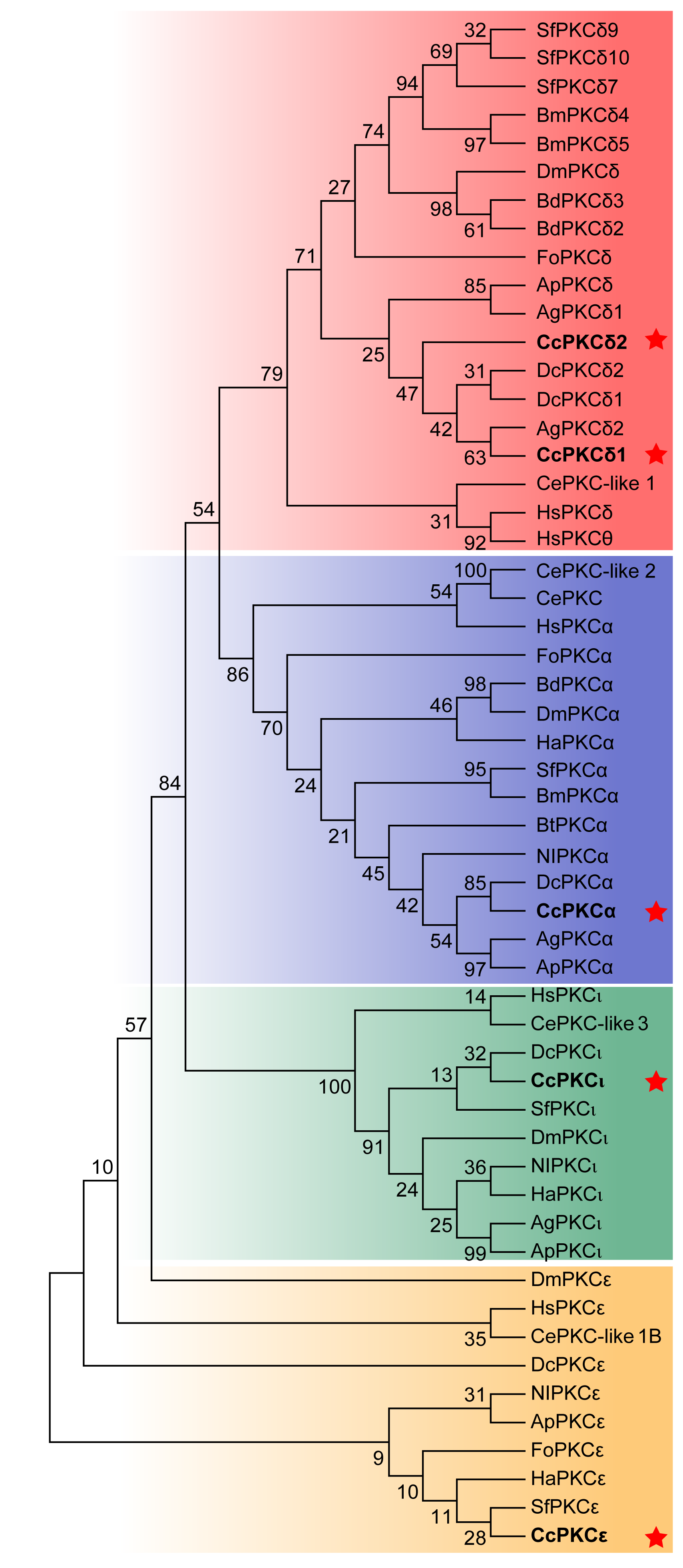


**Figure S10.** **Evolutionary relationships of PKC isoforms in insects.**

Phylogenetic tree of CcPKCα, CcPKCδ1, CcPKCδ2, CcPKCε, and CcPKCι (red stars) with orthologs from other species. Accession numbers in Table S2.


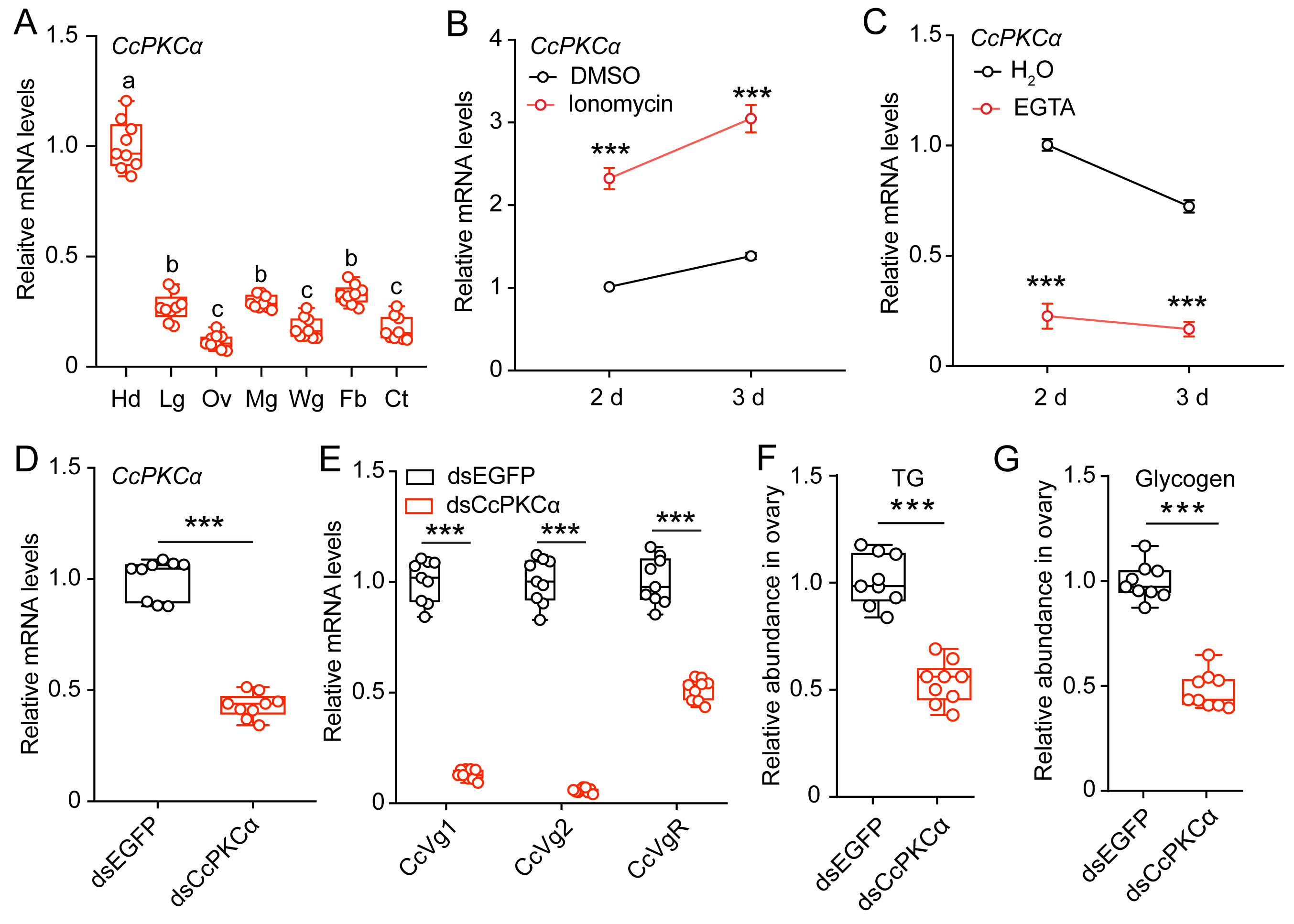


**Figure S11. Functional characterization of CcPKCα in vitellogenesis regulation.**

A: Tissue-specific *CcPKCα* expression (qRT-PCR): Hd (head), Lg (leg), Ov (ovary), Mg (midgut), Wg (wings), Fb (fat body), and Ct (cuticle). B-C: *CcPKCα* modulation by Ionomycin (1 μM in 0.1% DMSO) or EGTA (50 nM aqueous). D: RNAi efficiency of *CcPKCα* in winter-form females following dsRNA administration for 3 days under 25 °C conditions by qRT-PCR. E: The expression levels of *CcVg1*, *CcVg2,* and *CcVgR* post dsCcPKCα and dsEGFP treatments for 3 days. F-G: Impact of dsCcPKCα treatments on triglyceride levels and glycogen content. Data presentation of A-E: Mean ± SEM (n=3 biological × 3 technical replicates) and F-G: Mean ± SEM (n=9 biological replicates). Letters indicate significance (ANOVA/ Tukey’s HSD, *p* < 0.05). ****p* < 0.001(student’s *t*-test).

**
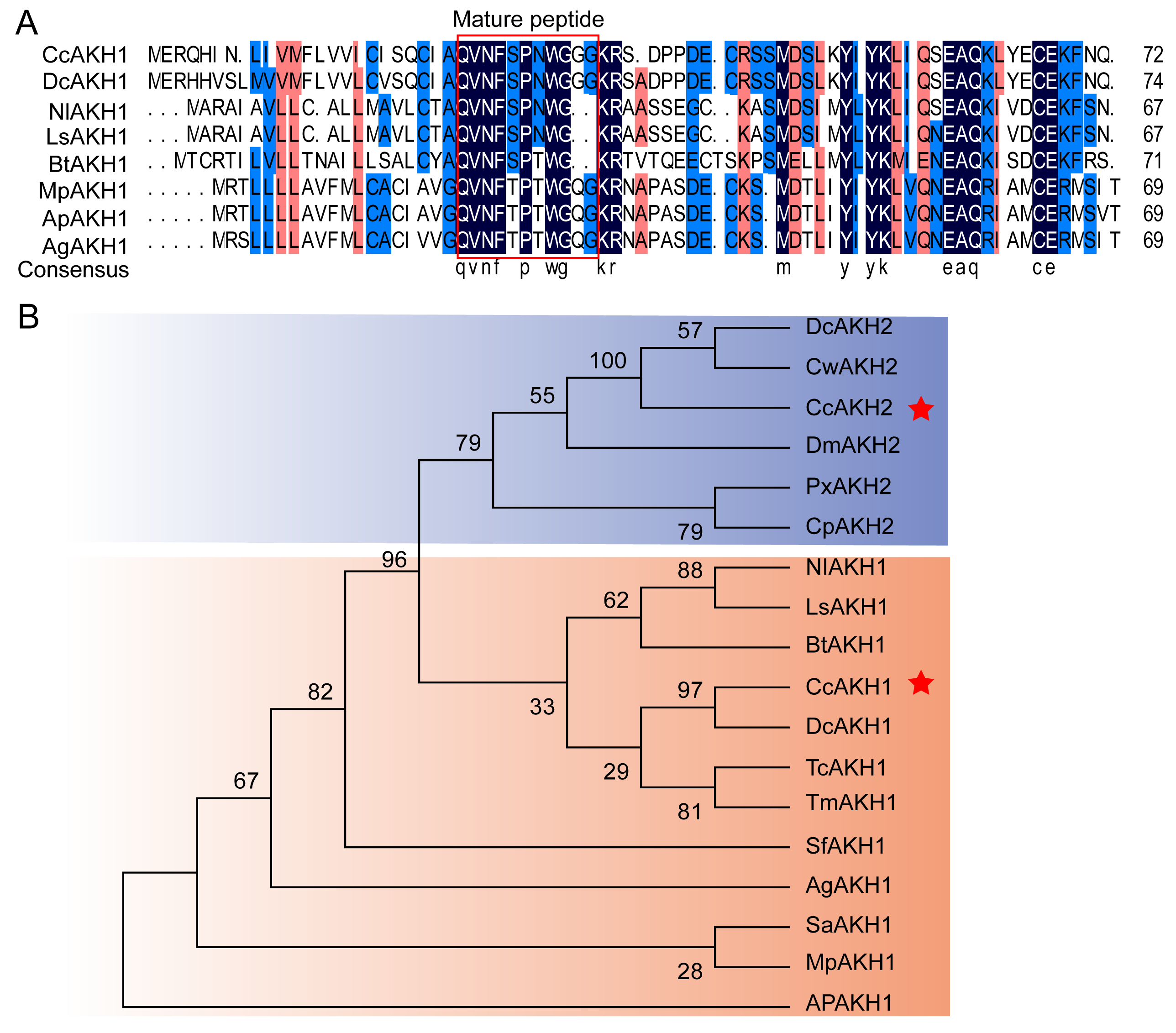
 Figure S12. Comparative analysis of AKH neuropeptides.**

A: Multiple alignments of CcAKH1 mature peptide (red box) with seven orthologs. *CcAKH1* (*C. chinensis*, PQ818816), *DcAKH1* (*D. citri*, XP_008488257.1), *NlAKH1* (*N. lugens*, AFN26934.1), *LsAKH1* (*L. striatellus*, AXF48182.1), *BtAKH1* (*B. tabaci*, XP_018896272.1), *MpAKH1* (*M. persicae*, XP_022166997.1), *ApAKH1* (*A. pisum*, NP_001243520.1), *AgAKH1* (*A. gossypii*, ALH44119.1). B: Phylogeny of CcAKH1 and CcAKH2 (red stars) with homologs (accession numbers in Table S2).


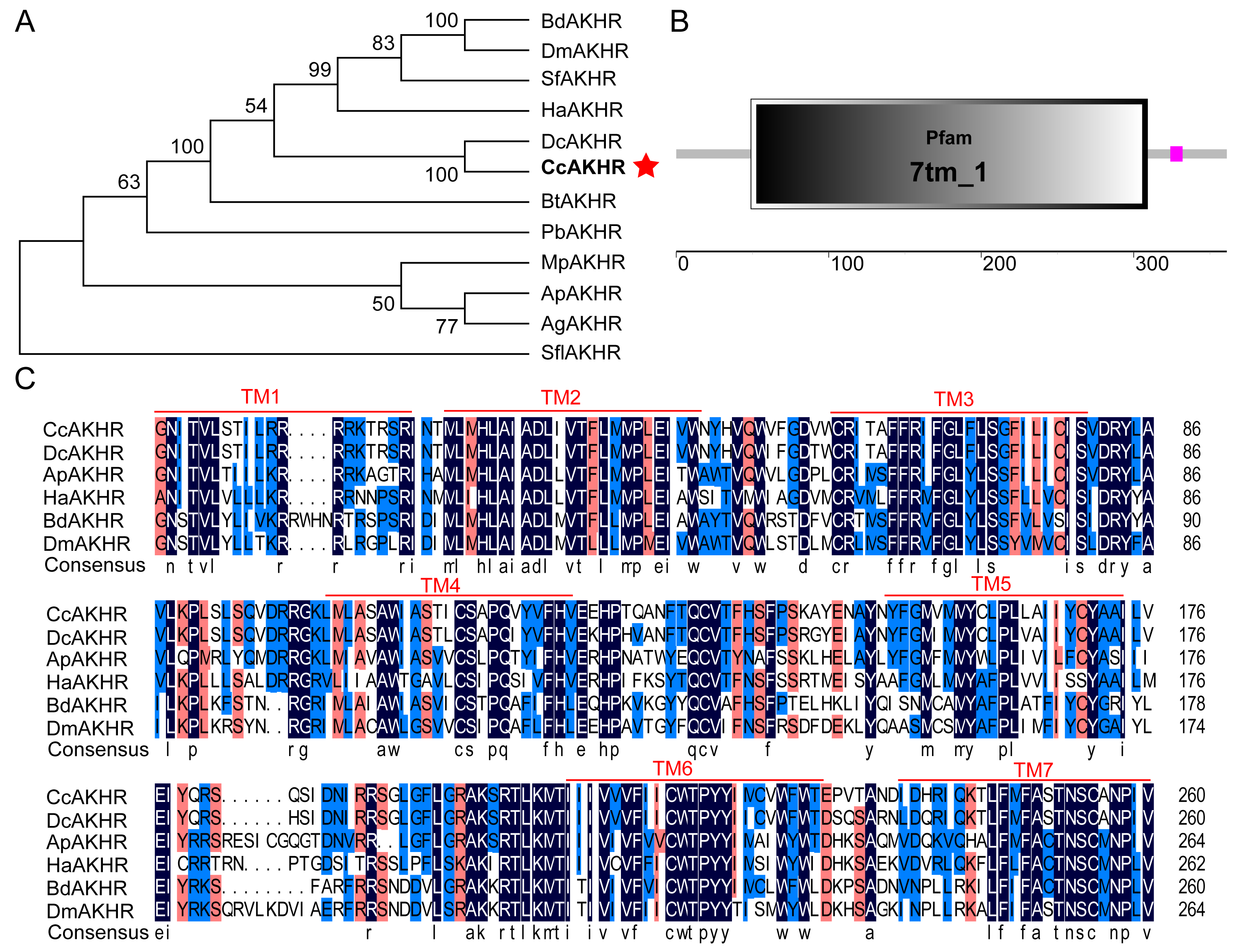


**Figure S13. Structural and evolutionary analysis of CcAKHR.**

A: Phylogenetic tree with CcAKHR (red star) and orthologs (Table S2). B: SMART domain architecture prediction. C: Transmembrane domain conservation (TM1-TM7, red bars) across six species. *CcAKHR* (*C. chinensis*, PQ818818), *DcAKHR* (*D. citri*, AWT50656.1), *ApAKHR* (*A. pisum*, XP_029343785.1), *HaAKHR* (*H. axyridis*, XP_045482662.1), *BdAKHR* (*B. dorsalis*, AQX83416.1), and *DmAKHR* (*D. melanogaster*, AAN10047.1).


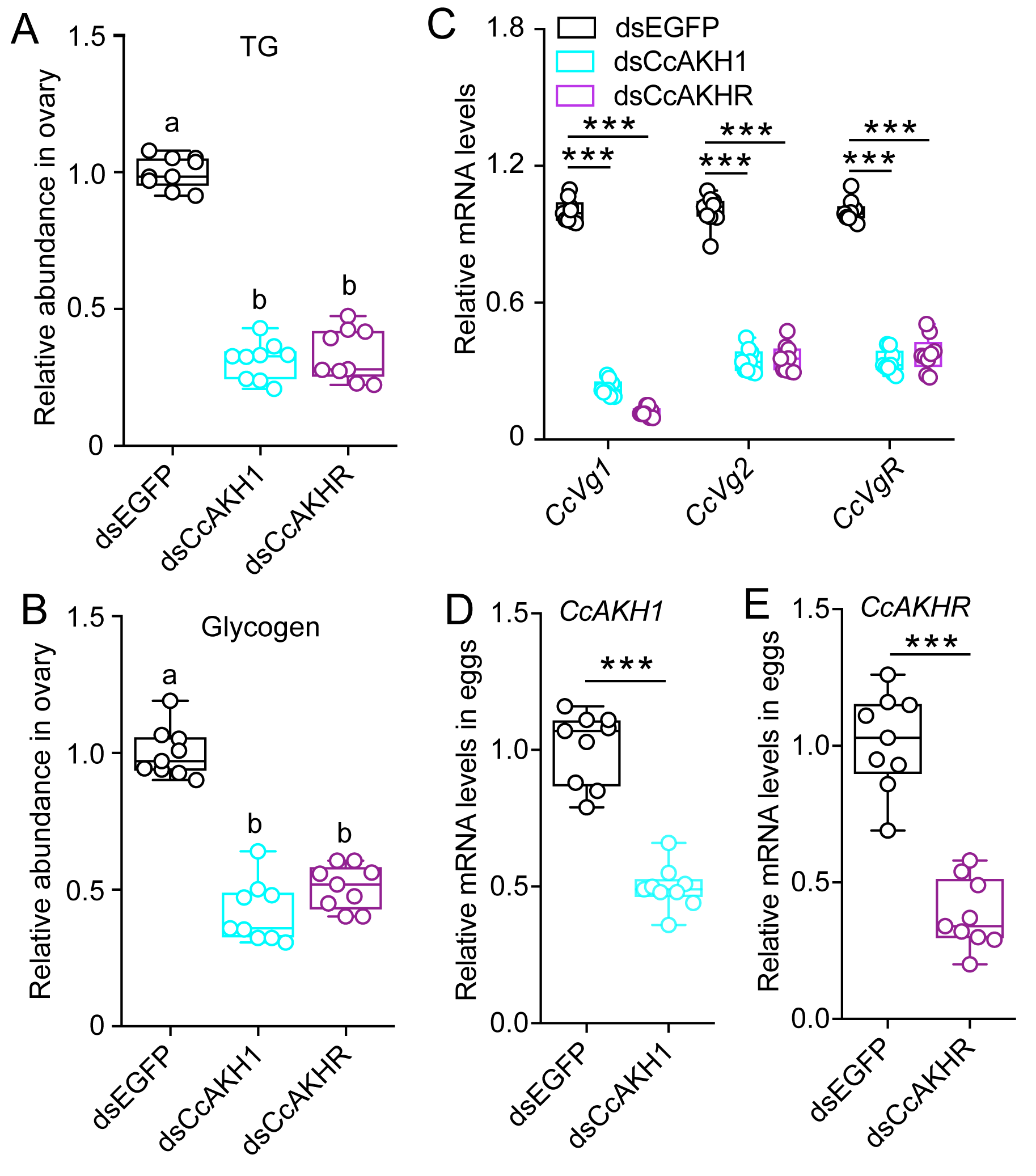


**Figure S14. Influences of dsCcAKH1 and dsCcAKHR treatments on triglyceride levels, glycogen content, mRNA expression of *CcVg1*, *CcVg2*, and *CcVgR*.**

Data presentation of A-B: Mean ± SEM (n=9 biological replicates) and C-E: Mean ± SEM (n=3 biological × 3 technical replicates). Statistical significance between groups was determined by pair-wise Student’s *t*-test (****p*<0.001; n.s.: no significant, *p*>0.05) in Graphpad Prism 8.0 software. Multiple comparisons: letters denote significant differences (ANOVA/Turkey’s HSD, *p*<0.05).


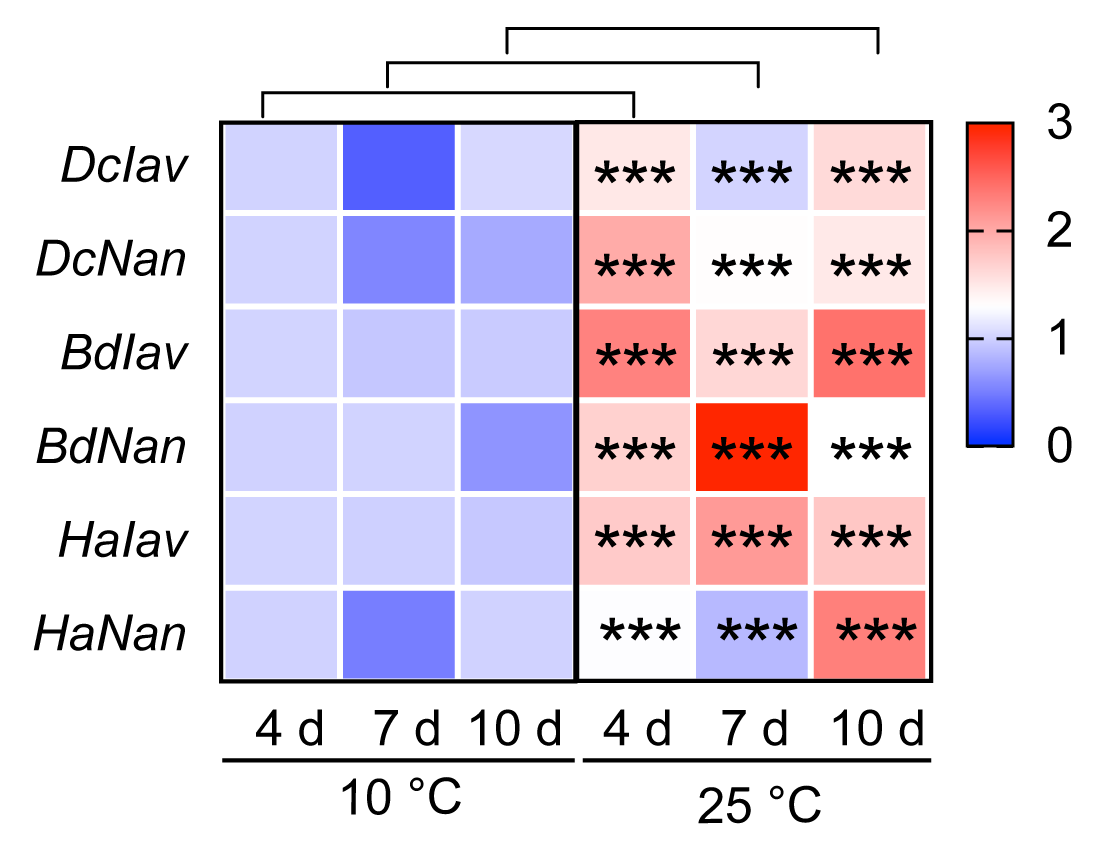


**Figure S15. Thermosensitive expression profiles of TRPV subunits across three insect species under 10 °C and 25 °C treatments at 4, 7, and 10 days post-exposure.**

Data presentation of mean (n= 3 biological × 3 technical replicates) and C-E: Mean ± SEM (n=3 biological × 3 technical replicates). Statistical significance (****p*<0.001) between groups was determined using the pair-wise Student’s *t*-test in Graphpad Prism 8.0 software.


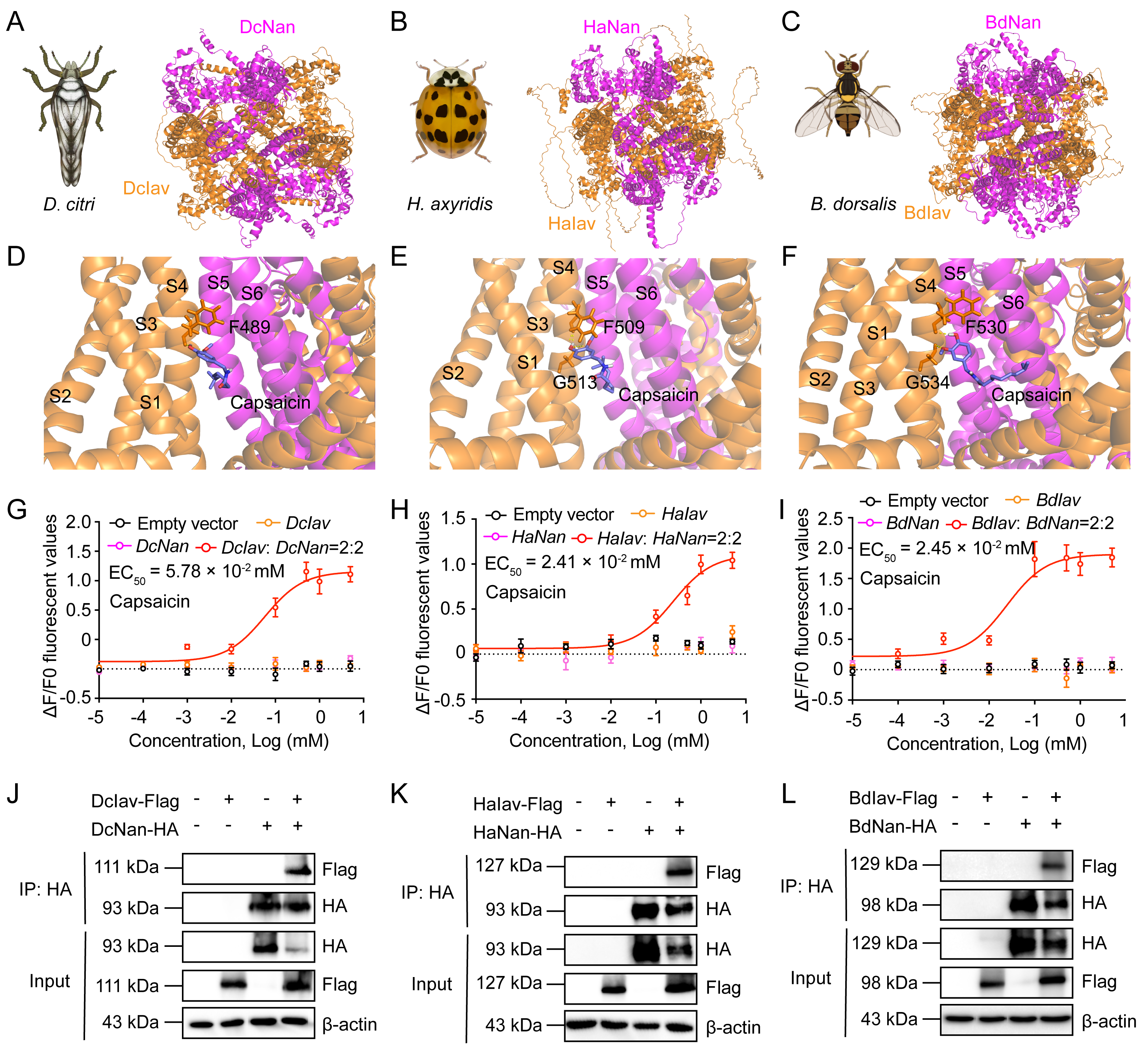


**Figure S16. Evolutionarily conserved TRPV heterotetramer function in thermosensation across three insect species.**

A-C: Predicted tertiary structures of Iav-Nan heterotetramers (2:2 ratio) in *D. citri*, *H. axyridis*, and *B. doralis* generated using AlphaFold3. D-F: Molecular docking of Iav-Nan (2:2) complex with capsaicin in *D. citri*, *H. axyridis*, and *B. doralis* using Proteins Plus and AutoDock 4.2.6 software. G-I: Dose-response curves of capsaicin-induced calcium flux (fluo-4 signals) in HEK293T cells expressing heterologously expressed Iav and Nan from *D. citri*, *H. axyridis*, and *B.doralis*. Recombinant vectors were generated by inserting the full-length coding sequences of Iav or Nan into the pcDNA3.1(+)-mCherry vector. An empty vector served as the negative control. Data: mean ± SEM (n=6). EC_50_ values derived from fitted curve. J-L: Co-IP verification of Iav-Nan interactions in *D. citri*, *H. axyridis*, and *B.doralis*. Flag-tagged Iav and/or HA-tagged Nan were introduced into HEK293T cells, followed by immunoprecipitation with anti-Flag and anti-HA antibody, separately.

**Table S1. The primers used in this study.**

| **Gene name**  **and accession number** | **Sequences of primers (5′–3′)** | **Product length (bp)** | **Application purpose** |
| --- | --- | --- | --- |
| *CcIav*  (PQ818806) | Full-F1: ATGGGGTCTTTGTGTTGTGG | 3003 | Full-length cDNA cloning |
|  | Full-R1: CTACATGCTATCTTGATCACT |  |  |
|  | qF1: CTGAGGCCAGTGATAGTGAT | 134 | qPCR |
|  | qR1: GGACTTGACCAATTTCTGTT |  |  |
|  | RNAi-F1: GATCACTAATACGACTCACTATAGGGAGATCCTTGAAGAGAAATGGAAA | 407 | dsRNA synthesis |
|  | RNAi-R1: GATCACTAATACGACTCACTATAGGGAGACATCAAGAGAAACCAAGAGC |  |  |
|  | pCDNA3.1-mcherry-Flag-F1: CCACTAGTCCAGTGTGGTGGAATTCGGCCACCATGGGGTCTTTGTGTTGTGG | 3078 | Calcium imaging and Western blot (pCDNA3.1-mcherry-P2A) |
|  | pCDNA3.1-mcherry-Flag-R1: GTTTAAACGGGCCCTCTAGACTACTTGTCATCGTCATCCTTGTAGTCGATGTCATGATCTTTATAATCACCGTCATGGTCTTTGTAGTCCTCGAGCATGCTATCTTGATCACTAG |  |  |
|  | pCMV-Flag-F1: ACTTAAGCTTGGTACCGAGCTCGGATCCGCCACCATGGGGTCTTTGTGTTG | 3003 | Subcellular co-localization (pCMC-SV40-Neo) |
|  | pCMV-Flag-R1: TCACCGTCATGGTCTTTGTAGTCCTCGAGCATGCTATCTTGATCACTAG |  |  |
|  | G511A-F1: TAATATTTGCTGTGCCTGCTTCTTGGTTTCTCTTGATGTT | 3003 | Site-directed mutagenesis |
|  | G511A-R1: AGCAGGCACAGCAAATATTAATATAGCTTCTTCTGTTTTCTTATCACC |  |  |
|  | F507A-F1: AAGAAGCTATATTAATAGCTGCTGTGCCTGGTTCTTGGTT | 3003 |  |
|  | F507A-R1: GCTATTAATATAGCTTCTTCTGTTTTCTTATCACCTATGATACGA |  |  |
| *CcNan*  (PQ818807) | Full-F1: ATGGGTAACACAGAGAGCAATGT | 2925 | Full-length cDNA cloning |
|  | Full-R1: TTTCAACAAAGGTATAGGTG |  |  |
|  | qF1: GGGTTGACGACACTGTTAGT | 126 | qPCR |
|  | qR1: CTACTGCATGTGCTGTTGTT |  |  |
|  | RNAi-F1: GATCACTAATACGACTCACTATAGGGAGAGGAGTGCTTTTGTCAAGTTC | 373 | dsRNA synthesis |
|  | RNAi-R1: GATCACTAATACGACTCACTATAGGGAGAATTTTCGATGGTGTATCAGC |  |  |
|  | pCDNA3.1-mcherry-HA-F1:  CCACTAGTCCAGTGTGGTGGAATTCGGCCACCATGGGTAACACAGAGAGCAATGT | 3030 | Calcium imaging and Western blot (pCDNA3.1-mcherry-P2A) |
|  | pCDNA3.1-mcherry-HA-R1: GTTTAAACGGGCCCTCTAGACTACTGAGCAGCGTAATCTGGAACGTCATATGGATAGGACCCTGCATAGTCCGGGACGTCATAGGGATAGCCCGCATAGTCAGGAACATCGTATGGGTACTCGAGTTTCAACAAAGGTATAGGTG |  |  |
|  | pCMV-HA-F1 ACTTAAGCTTGGTACCGAGCTCGGATCCGCCACCATGGGTAACACAGAGAGCAA | 3030 | Subcellular co-localization (pCMC-SV40-Neo) |
|  | pCMV-HA-R1: TCACCGTCATGGTCTTTGTAGTCCTCGAGCTACTGAGCAGCGTAATCTG |  |  |
| *CcTRPA1*  (PP096834) | qF1: CTGATTGGTTTGGCTGTC | 150 | qPCR |
|  | qR1: CTCGTAGATTTCCATTTTGTC |  |  |
| *CcPyrexia*  (PQ818808) | qF1: AGGCAGAGGAGAGTAGATCC | 132 | qPCR |
|  | qR1: ATTCAAAGTGTTTTGTTGTTCA |  |  |
| *CcPainless*  (PQ818809) | qF1: GCAACTCTTATGGTTCTCGT | 132 | qPCR |
|  | qR1: TGGTGACAATCTTGAGCAT |  |  |
| *CcPainless-like*  (PQ818810) | qF1: ATGGACAAGGAGATCATACG | 129 | qPCR |
|  | qR1: ATGTTCCAGCTTGTTCTGAT |  |  |
| *CcPKCα*  (PQ818811) | Full-F1: ATGGCCGACGAAGAGAATAGCAACGAA | 2007 | Full-length cDNA cloning |
|  | Full-R1: GACATGTTGAATAAACTCTGGATTC |  |  |
|  | qF1: TCCTCGAGAAGAAGAAGATG | 145 | qPCR |
|  | qR1: GGTGAATTGTTTGTCGAAAT |  |  |
|  | RNAi-F1: GATCACTAATACGACTCACTATAGGGAGATTCAATGTCAAGTGTGCAGT | 392 | dsRNA synthesis |
|  | RNAi-R1: GATCACTAATACGACTCACTATAGGGAGAGTACGGATCTGATTGACCAT |  |  |
|  | pCDNA3.1-mcherry-V5-His-F1: CCACTAGTCCAGTGTGGTGGAATTCGGCCACCATGGCCGACGAAGAGAATAGC | 2076 | Western blot (pCDNA3.1-mcherry-P2A) |
|  | pCDNA3.1-mcherry-V5-His-R1：GTTTAAACGGGCCCTCTAGACATGGTGATGGTGATGATGACCGGTACGCGTAGAATCGAGACCGAGGAGAGGGTTAGGGATAGGCTTACCGACATGTTGAATAAACTCTGGATTC |  |  |
| *CcPKCδ1*  (PQ818812) | qF1: CGACCATTGTGGATCTCTAT | 148 | qPCR |
|  | qR1: AGAGGTTAACGCTTCTACGA |  |  |
| *CcPKCδ2*  (PQ818813) | qF1: TTCAGTGGTTGTGATGAAGA | 132 | qPCR |
|  | qR1: CAGTCTTTTGGTTGTGTCCT |  |  |
| *CcPKCε*  (PQ818814) | qF1: GTTACGTTAGCCTTGCAGTT | 124 | qPCR |
|  | qR1: CCTCCTTACACATTCCAAAG |  |  |
| *CcPKCι*  (PQ818815) | qF1: GTTGGAAGCAAAACAAGTTC | 145 | qPCR |
|  | qR1: AGACTGGTCGATTTTCTCAA |  |  |
| *CcAKH1*  (PQ818816) | Full-F1: ATGGAGCGCCAGCACATAA | 219 | Full-length cDNA cloning |
|  | Full-R1: TTACTGGTTGAACTTTTCAC |  |  |
|  | qF1: GAAAGAGATCTGACCCTCCT | 102 | qPCR |
|  | qR1: CACATTCGTACAACTTTTGG |  |  |
|  | RNAi-F1: GATCACTAATACGACTCACTATAGGGAGAGCCAGCACATAAATCTCATA | 160 | dsRNA synthesis |
|  | RNAi-R1: GATCACTAATACGACTCACTATAGGGAGAAGGAGGGTCAGATCTCTTTC |  |  |
| *CcAKH2*  (PQ818817) | Full-F1: ATGTTGAAAATTTTCTTCAT | 330 | Full-length cDNA cloning |
|  | Full-R1: CTAGTGCACTTTGTCCTCTT |  |  |
|  | qF1: GGGTATACGAAACTTGCAGA | 103 | qPCR |
|  | qR1: CATCAAGTCCTGCTTCTGAT |  |  |
| *CcAKHR*  (PQ818818) | Full-F1: ATGGCGCTGACAACTGAT | 1086 | Full-length cDNA cloning |
|  | Full-R1: CTAATACTCCTCTGTCCGTA |  |  |
|  | qF1: CATTGTGTACGGATTGTTCA | 109 | qPCR |
|  | qR1: ATAACTTGCAACCGAAGCTA |  |  |
|  | RNAi-F1: GATCACTAATACGACTCACTATAGGGAGACTTTCTAATGATGCCTCTGG | 298 | dsRNA synthesis |
|  | RNAi-R1: GATCACTAATACGACTCACTATAGGGAGAGGTAAAGTTAGCCTGTGTGG |  |  |
|  | pCDNA3.1-mcherry-Myc-His-F1:  CCACTAGTCCAGTGTGGTGGAATTCGGCCACCATGGCGCTGACAACTGAT | 1152 | Western blot (pCDNA3.1-mcherry-P2A) |
|  | pCDNA3.1-mcherry-Myc-His-R1: GTTTAAACGGGCCCTCTAGAATGATGATGATGATGATGGTCGACGGCGCTATTCAGATCCTCTTCTGAGATGAGTTTTTGTTCCTCGAGATACTCCTCTGTCCGTATCG |  |  |
|  | pEGFP-F1: CGCTAGCGCTACCGGACTCAGATCTGCCACCATGGCGCTGACAACTGAT | 1086 | Subcellular localization (pEGFP-3×linker) |
|  | pEGFP-R1: CTCCACTCCCTCCGCCGCCGGTACCATACTCCTCTGTCCGTA |  |  |
|  | T64D-F1: TGCGGCGTAGGAGGAAAGATCGTTCGAGGATCAACACCAT | 1086 | Site-directed mutagenesis |
|  | T64D-R1: ATCTTTCCTCCTACGCCGCAAGATGGTGCTCAACACTGTA |  |  |
|  | T251D-F1: GAAGGGCAAAGTCTCGGGATCTAAAGATGACTATTATAAT |  |  |
|  | T251D-R1: ATCCCGAGACTTTGCCCTTCCTAGGAACCCAAGGCCG |  |  |
|  | S326D-F1: CAAATCAGGTAGGCGGCGATCGAGGAGGCAGGGGGGGCAA |  |  |
|  | S326D-R1: ATCGCCGCCTACCTGATTTGCCCCCCTTCTTGTCTTTATG |  |  |
|  | S338D-F1: GCAAGAAATGTTTAGCTGATGTTGCAAGTTATTCAAGTGG |  |  |
|  | S338D-R1: ATCAGCTAAACATTTCTTGCCCCCCCTGCCTCCTCG |  |  |
|  | T64A-F1: TGCGGCGTAGGAGGAAAGCCCGTTCGAGGATCAACACCAT |  |  |
|  | T64A-R1: GGCTTTCCTCCTACGCCGCAAGATGGTGCTCAACACTGTA |  |  |
|  | T251A-F1: GAAGGGCAAAGTCTCGGGCCCTAAAGATGACTATTATAAT |  |  |
|  | T251A-R1: GGCCCGAGACTTTGCCCTTCCTAGGAACCCAAGGCCGG |  |  |
|  | S326A-F1: CAAATCAGGTAGGCGGCGCCCGAGGAGGCAGGGGGGGCAA |  |  |
|  | S326A-R1: GGCGCCGCCTACCTGATTTGCCCCCCTTCTTGTCTTTATG |  |  |
|  | S338A-F1: AAATGTTTAGCTGCCGTTGCAAGTTATTCAAGTGG |  |  |
|  | S338A-R1: GGCAGCTAAACATTTCTTGCCCCCCCTGCCTCCTCG |  |  |
| *DcIav*  (PQ818822) | Full-F1: ATGAATGTCGGTTCCGTTTTA | 2976 | Full-length cDNA cloning |
|  | Full-R1: CATGCTATCTTGATCACTAGG |  |  |
|  | qF1: AGTAGTTCGAGCTCCTCTCC | 137 | qPCR |
|  | qR1: TCAGGTGTAGGAGCAACTCT |  |  |
|  | pCDNA3.1-mcherry-Flag-F1: CCACTAGTCCAGTGTGGTGGAATTCGGCCACCATGAATGTCGGTTCCGTTTTAGATCGAGTG | 2976 | Calcium imaging and Western blot (pCDNA3.1-mcherry-P2A) |
|  | pCDNA3.1-mcherry-Flag-R1: ATCACCGTCATGGTCTTTGTAGTCCTCGAGCATGCTATCTTGATCACTAGG |  |  |
| *DCNan*  (PQ818823) | Full-F1: ATGGGAAACACAGAGAGCAATGT | 2439 | Full-length cDNA cloning |
|  | Full-R1: TTTGAGAAGGGGTGAAGCTTTTGCTAAGTT |  |  |
|  | qF1: CCGAGACTAGAAATGAGTGG | 149 | qPCR |
|  | qR1: GTTGAGTCGAAGGACAAGAG |  |  |
|  | pCDNA3.1-mcherry-HA-F1: CCACTAGTCCAGTGTGGTGGAATTCGGCCACCATGGGAAACACAGAGAGCAATGT | 2439 | Calcium imaging and Western blot (pCDNA3.1-mcherry-P2A) |
|  | pCDNA3.1-mcherry-HA-R1: ATAGTCAGGAACATCGTATGGGTACTCGAG TTTGAGAAGGGGTGAAGCTTTTGCTAAGTT |  |  |
| *BdIav*  (PQ818824) | Full-F1: ATGAAATTCTTTTTGAAAAAATG | 3477 | Full-length cDNA cloning |
|  | Full-R1: CTTTCGCGCCAATATTTTGTTGATATT |  |  |
|  | qF1: CATACACTCACCATCGAACA | 121 | qPCR |
|  | qR1: GCAGGTGAAATTGTTTCATT |  |  |
|  | pCDNA3.1-mcherry-Flag-F1: CCACTAGTCCAGTGTGGTGGAATTCGGCCACCATGAAGCCCGAAGAGATGAAA | 3477 | Calcium imaging and Western blot (pCDNA3.1-mcherry-P2A) |
|  | pCDNA3.1-mcherry-Flag-R1: ATCACCGTCATGGTCTTTGTAGTCCTCGAGCTTTCGCGCCAATATTTTGTTGATATT |  |  |
| *BdNan*  (PQ818825) | Full-F1: ATGGGGAATACGGAGAGCAA | 2538 | Full-length cDNA cloning |
|  | Full-R1: AGTATTATTATTATCACGGTCACTC |  |  |
|  | qF1: CTGGTAGTGGAGCGTAGTGT | 130 | qPCR |
|  | qR1: TCTCTTCTTTGTCCTCATCG |  |  |
|  | pCDNA3.1-mcherry-HA-F1: CCACTAGTCCAGTGTGGTGGAATTCGGCCACCATGGGGAATACGGAGAGCAA | 2538 | Calcium imaging and Western blot (pCDNA3.1-mcherry-P2A) |
|  | pCDNA3.1-mcherry-HA-R1: ATAGTCAGGAACATCGTATGGGTACTCGAGAGTATTATTATTATCACGGTCACTC |  |  |
| *HaIav*  (PQ818826) | Full-F1: ATGGGAGCATGTAACTGCAAAGT | 3405 | Full-length cDNA cloning |
|  | Full-R1: CATGCTATCATGTTCATTTTCCC |  |  |
|  | qF1: GGTCATCCTAGTCAATCAGG | 131 | qPCR |
|  | qR1: TCGGAGTTCAATGGTTTACT |  |  |
|  | pCDNA3.1-mcherry-Flag-F1: CCACTAGTCCAGTGTGGTGGAATTCGGCCACCATGGGAGCATGTAACTGCAAAGT | 3405 | Calcium imaging and Western blot (pCDNA3.1-mcherry-P2A) |
|  | pCDNA3.1-mcherry-Flag-R1: ATCACCGTCATGGTCTTTGTAGTCCTCGAGCATGCTATCATGTTCATTTTCCC |  |  |
| *HaNan*  (PQ818827) | Full-F1: ATGGGAAATACAGAAAGTAA | 2412 | Full-length cDNA cloning |
|  | Full-R1: AACCATTTTGTTCAGTATTCCCT |  |  |
|  | qF1: TAACGAATTTTGGCGACTAT | 144 | qPCR |
|  | qR1: TTTGATAAGTGTTCCCCATC |  |  |
|  | pCDNA3.1-mcherry-HA-F1: CCACTAGTCCAGTGTGGTGGAATTCGGCCACCATGGGAAATACAGAAAGTAATGTCA | 2412 | Calcium imaging and Western blot (pCDNA3.1-mcherry-P2A) |
|  | pCDNA3.1-mcherry-HA-R1: ATAGTCAGGAACATCGTATGGGTACTCGAGAACCATTTTGTTCAGTATTCCCT |  |  |
| *CcVg1*  (PQ818819) | qF1: ATGCACCTGTTTCTTCTGAA | 140 | qPCR |
|  | qR1: CATACGAGAGCTGGACATAG |  |  |
| *CcVg2*  (PQ818820) | qF1: CTACGATGCCCAAAGATCAT | 126 | qPCR |
|  | qR1: TTGTTGGAATTGTTGGCATC |  |  |
| *CcVgR]* (PQ818821) | qF1: TAAGGGGAAGAAGTCAAGGA | 146 | qPCR |
|  | qR1: AGACATCTCATTGGCAAACT |  |  |
| *Ccβ-actin*  (OQ658571) | qF1: CTTTCACCACCACCGCTG | 154 | qPCR |
|  | qR1: CGCAAGATTCCATACCCA |  |  |
| *CcEF1*  (OQ658572) | qF1: CAATGTTGGTTTCAACGTAA | 130 | qPCR |
|  | qR1: GTGGTTCAATACGATGACCT |  |  |
| *Dcβ-actin*  (DQ675553.1） | qF1: TGTTCCAACCTTCCTTCCTG | 109 | qPCR |
|  | qR1: GTGTTGGCGTACAGGTCCTT |  |  |
| *DcEF1*  (XM_008479903) | qF1: CTGGGAGTGAAACAACTCAT | 127 | qPCR |
|  | qR1: GGTTGTAACCGATCTTCTTG |  |  |
| *Bdβ-actin* (HQ585895.1) | qF1: TGAACACGGTATTGTAACCA | 147 | qPCR |
|  | qR1: GTCATCTTCTCACGGTTAGC |  |  |
| *BdEF1*  (GU339154.1) | qF1: TGCGTACTAACGACGATAAA | 129 | qPCR |
|  | qR1: CTCCGTTTTCCTTACTGTTG |  |  |
| *Haβ-actin*  (MG983770.1) | qF1: TGGGACAAAAGGACTCATAC | 144 | qPCR |
|  | qR1: GGAGTTCGTTGTAGAAGGTG |  |  |
| *HaEF1*  (MG983771.1) | qF1: AAATCAGCAACGGATACACT | 138 | qPCR |
|  | qR1: CTCCAGATTTGATAGCCTTG |  |  |
| *EGFP*  （ACY56286） | RNAi-F1:  GATCACTAATACGACTCACTATAGGGAGA ACTCCAGCAGGACCATGTGATC | 596 | dsRNA synthesis |
|  | RNAi-R1:  GATCACTAATACGACTCACTATAGGGAGAACCTGAAGTTCATCTGCACCAC |  |  |

Note: The black boxes showed the T7 promoter sequences in the primers of dsRNA synthesis. The underline indicated the homologous arm sequence used for vector seamless clone. The dashed line represented the increased Tag protein sequence.

**Table S2. The accession number of gene sequences used in sequence analysis.**

| **Species name** | **Gene Name** | **Accession number** |
| --- | --- | --- |
| *Acyrthosiphon pisum* | ApIav | XP_001950096.1 |
| *Aphis gossypii* | AgIav | XP_027842975.2 |
| *Bombyx mori* | BmIav | XP_004925321.1 |
| *Frankliniella occidentalis* | FoIav | XP_026273275.1 |
| *Spodoptera frugiperda* | SfIav | XP_035451901.2 |
| *Bemisia tabaci* | BtIav | WMY99267.1 |
| *Drosophila melanogaster* | DmIav | NP_572353.1 |
| *Nilaparvata lugens* | NlIav | AOR81475.1 |
| *Acyrthosiphon pisum* | ApNan | XP_016658292.1 |
| *Aphis gossypii* | AgNan | XP_050054408.1 |
| *Bemisia tabaci* | BtNan | WMY99268.1 |
| *Bombyx mori* | BmNan | XP_062528665.1 |
| *Drosophila melanogaster* | DmNan | NP_648696.2 |
| *Nilaparvata lugens* | NlNan | AOR81474.1 |
| *Spodoptera frugiperda* | SfNan | XP_035430641.1 |
| *Frankliniella occidentalis* | FoNan | XP_052132718.1 |
| *Caenorhabditis elegans* | CeOcr-1 | NP_001335506.1 |
| *Caenorhabditis elegans* | CeOcr-2 | NP_501380.1 |
| *Caenorhabditis elegans* | CeOcr-3 | NP_510520.4 |
| *Caenorhabditis elegans* | CeOcr-4 | NP_501172.2 |
| *Caenorhabditis elegans* | CeOSM9 | ACQ44032.1 |
| *Homo sapiens* | HsTRPV1 | NP_061197.4 |
| *Homo sapiens* | HsTRPV2 | NP_057197.2 |
| *Mus musculus* | MmTRPV3 | AAI08985.1 |
| *Homo sapiens* | HsTRPV5 | NP_062815.3 |
| *Mus musculus* | MmTRPV5 | NP_001007573.1 |
| *Mus musculus* | MmTRPV6 | NP_071858.3 |
| Homo sapiens | HsTRPV6 | CAC20416.2 |
| *Caenorhabditis elegans* | CePKC | NP_001360727.1 |
| *Caenorhabditis elegans* | CePKC-like 1 | NP_001359809.1 |
| *Caenorhabditis elegans* | CePKC-like 1B | NP_001256394.1 |
| *Caenorhabditis elegans* | CePKC-like 2 | NP_001024516.1 |
| *Caenorhabditis elegans* | CePKC-like 3 | NP_495011.1 |
| *Diaphorina citri* | DcPKCα | XP_026685144.1 |
| *Bemisia tabaci* | BtPKCα | XP_018903789.1 |
| *Aphis gossypii* | AgPKCα | XP_027848158.1 |
| *Acyrthosiphon pisum* | ApPKCα | XP_001945397.2 |
| *Nilaparvata lugens* | NlPKCα | XP_022194978.1 |
| *Frankliniella occidentalis* | FoPKCα | XP_026275736.1 |
| *Harmonia axyridis* | HaPKCα | XP_045465229.1 |
| *Spodoptera frugiperda* | SfPKCα | XP_035440495.1 |
| *Bombyx mori* | BmPKCα | NP_001036978.1 |
| *Bactrocera dorsalis* | BdPKCα | XP_049307882.1 |
| *Drosophila melanogaster* | DmPKCα | CAA28890.2 |
| *Homo sapiens* | HsPKCα | BAU98542.1 |
| *Drosophila melanogaster* | DmPKCδ | NP_001138191.1 |
| *Homo sapiens* | HsPKCδ | BAA01381.1 |
| *Frankliniella occidentalis* | FoPKCδ | XP_052126934.1 |
| *Acyrthosiphon pisum* | ApPKCδ | XP_001945932.2 |
| *Diaphorina citri* | DcPKCδ1 | XP_026682131.1 |
| *Aphis gossypii* | AgPKCδ1 | XP_027843850.2 |
| *Spodoptera frugiperda* | SfPKCδ10 | XP_050557356.1 |
| *Diaphorina citri* | DcPKCδ2 | XP_026682132.1 |
| *Bactrocera dorsalis* | BdPKCδ2 | XP_011206367.2 |
| *Aphis gossypii* | AgPKCδ2 | XP_050058289.1 |
| *Bactrocera dorsalis* | BdPKCδ3 | XP_019846577.2 |
| *Bombyx mori* | BmPKCδ4 | XP_037874771.1 |
| *Bombyx mori* | BmPKCδ5 | XP_062531002.1 |
| *Spodoptera frugiperda* | SfPKCδ7 | XP_050557353.1 |
| *Spodoptera frugiperda* | SfPKCδ9 | XP_050557355.1 |
| *Diaphorina citri* | DcPKCε | XP_026680683.1 |
| *Nilaparvata lugens* | NlPKCε | XP_022188143.1 |
| *Frankliniella occidentalis* | FoPKCε | XP_052133408.1 |
| *Spodoptera frugiperda* | SfPKCε | XP_035449834.1 |
| *Acyrthosiphon pisum* | ApPKCε | XP_001947622.2 |
| *Harmonia axyridis* | HaPKCε | XP_045483594.1 |
| *Drosophila melanogaster* | DmPKCε | NP_001163767.1 |
| *Homo sapiens* | HsPKCε | XP_011531282.1 |
| *Homo sapiens* | HsPKCθ | AAA75571.1 |
| *Diaphorina citri* | DcPKCι | XP_026683677.1 |
| *Nilaparvata lugens* | NlPKCι | XP_039300969.1 |
| *Aphis gossypii* | AgPKCι | XP_027846246.1 |
| *Harmonia axyridis* | HaPKCι | XP_045478348.1 |
| *Acyrthosiphon pisum* | ApPKCι | XP_008183802.2 |
| *Spodoptera frugiperda* | SfPKCι | XP_050549826.1 |
| *Drosophila melanogaster* | DmPKCι | AAG01528.1 |
| *Homo sapiens* | HsPKCι | AAA60171.1 |
| *Laodelphax striatellus* | LsAKH1 | AXF48182.1 |
| *Tenebrio molitor* | TmAKH1 | UXO98184.1 |
| *Sitobion avenae* | SaAKH1 | ALH44120.1 |
| *Myzus persicae* | MpAKH1 | XP_022166997.1 |
| *Sipha flava* | SflAKH1 | XP_025422856.1 |
| *Tribolium castaneum* | TcAKH1 | ABB58739.1 |
| *Diaphorina citri* | DcAKH1 | XP_008488257.1 |
| *Nilaparvata lugens* | NlAKH1 | AFN26934.1 |
| *Acyrthosiphon pisum* | ApAKH1 | NP_001243520.1 |
| *Aphis gossypii* | AgAKH1 | ALH44119.1 |
| *Bemisia tabaci* | BtAKH1 | XP_018896272.1 |
| *Drosophila melanogaster* | DmAKH1 | NP_523918.1 |
| *Cyamophila willieti* | CwAKH2 | KAL1461859.1 |
| *Diaphorina citri* | DcAKH2 | XP_008480698.1 |
| *Schistocerca gregaria* | SgAKH2 | UGX04160.1 |
| *Cydia pomonella* | CpAKH2 | QDK59876.1 |
| *Diaphorina citri* | DcAKHR | AWT50656.1 |
| *Acyrthosiphon pisum* | ApAKHR | XP_029343785.1 |
| *Aphis gossypii* | AgAKHR | XP_027836902.2 |
| *Bemisia tabaci* | BtAKHR | XP_018916522.1 |
| *Harmonia axyridis* | HaAKHR | XP_045482662.1 |
| *Bactrocera dorsalis* | BdAKHR | AQX83416.1 |
| *Spodoptera frugiperda* | SfAKHR | XP_035434918.1 |
| *Drosophila melanogaster* | DmAKHR | AAN10047.1 |
| *Sipha flava* | SflAKHR | XP_025424047.1 |
| *Pseudoregma bambucicola* | PbAKHR | AKH80288.1 |
| *Myzus persicae* | MpAKHR | XP_022164561.1 |
